# Supplementary material for: Individual Limb Muscle Bundles Are Formed through Progressive Steps Orchestrated by Adjacent Connective Tissue Cells during Primary Myogenesis
Source: Cell Rep. 2020 Mar 10;30(10):3552–3565.e6. doi: 10.1016/j.celrep.2020.02.037 (PMC7068676; doi:10.1016/j.celrep.2020.02.037)
Supplement: Document S1. Figures S1–S4 [file mmc1.pdf]

Cell Reports, Volume *30*

Supplemental Information

Individual Limb Muscle Bundles Are Formed through Progressive Steps Orchestrated by Adjacent Connective Tissue Cells during Primary Myogenesis

Laurianne Besse, Caroline J. Sheeba, Mark Holt, Maurice Labuhn, Susan Wilde, Eleanor Feneck, Donald Bell, Ania Kucharska, and Malcolm P.O. Logan

Supplemental Text and Figures

Individual limb muscle bundles are formed through progressive steps orchestrated by adjacent connective tissue cells during primary myogenesis.

Besse et al.,

Cell Reports D-17-03107

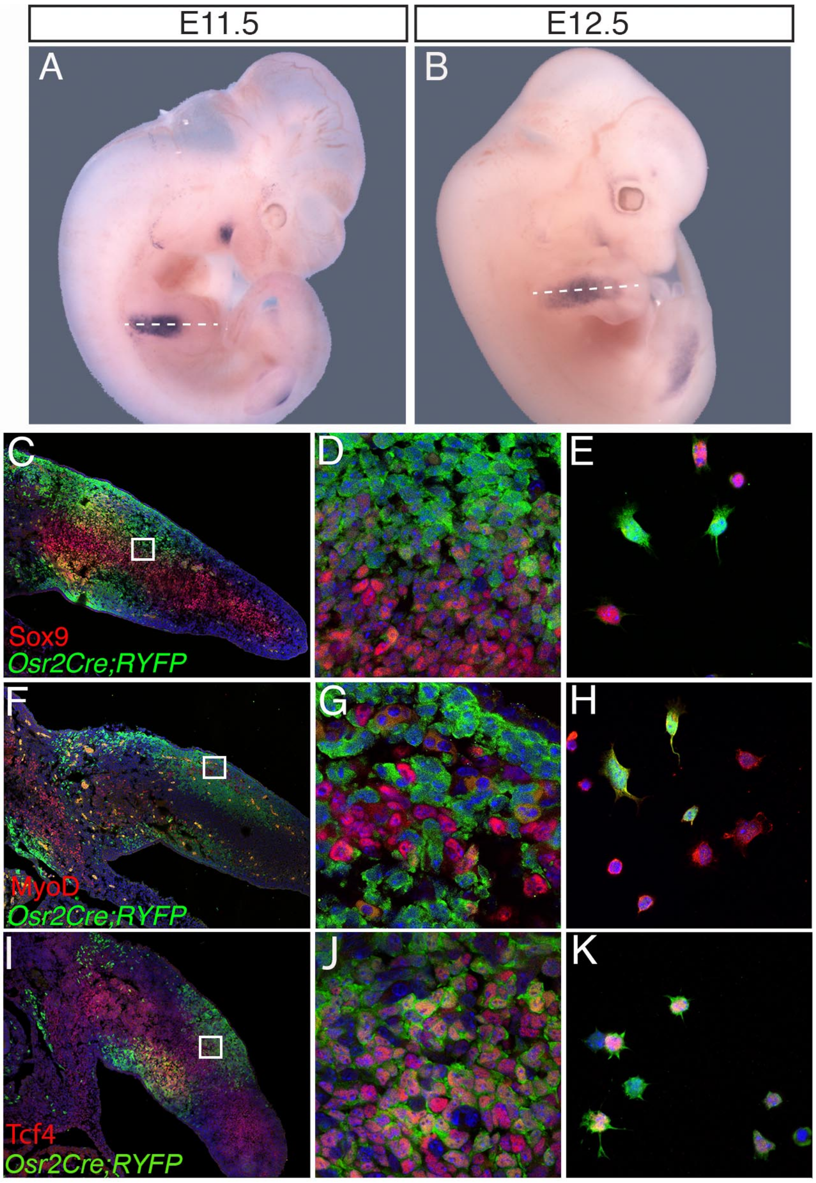

Figure S1. ***Osr2Cre*** **is active in forelimb MCT. Related to Figures 2, 3 and 4**
(A, B) Whole mount immunohistochemistry to detect alkaline phosphatase showing Cre activity almost exclusively restricted to the limbs at E11.5 (A) and E12.5 (B). Dashed lines show approximate plane of section shown in C,D, F,G, I,J. Immunohistochemistry on E11.5 *Osr2Cre;RosaYFP* forelimb cryosections to detect Sox9 (C-D), MyoD (F-G) and Tcf4 (I-J) (all red channel) also labelled for YFP (green) and DAPI (blue). The majority of *Osr2Cre* activity (YFP+) was not detected in the cartilage

cells (Sox9 +) or muscle progenitors (MyoD+) precursor cells. *Osr2Cre* activity (YFP+) overlaps with Tcf4+ MCT cells. The same immunohistochemical staining was carried out on dissociated cells in culture isolated from E11.5 limb buds (E, H, K show representative views of labelled cells). E. Of a total of 83 cells analysed, 36 were Sox9+ive only, 41 GFP(cre)+ive only and 6 were double labelled for both GFP(cre) and Sox9. H. Of a total of 431 cells analysed, 315 were MyoD +ive only, 116 were GFP/Cre +ive only and 0 were double-labelled for both GFP(cre) and MyoD. K. Of a total of 116 cells analysed, 5 were TCF+ive only, 46 were GFP/cre+ive only and 65 were double-labelled for TCF4/cre. Panels C,F,I are 20X, panels D-E, G-H and J-K are 100X magnification.

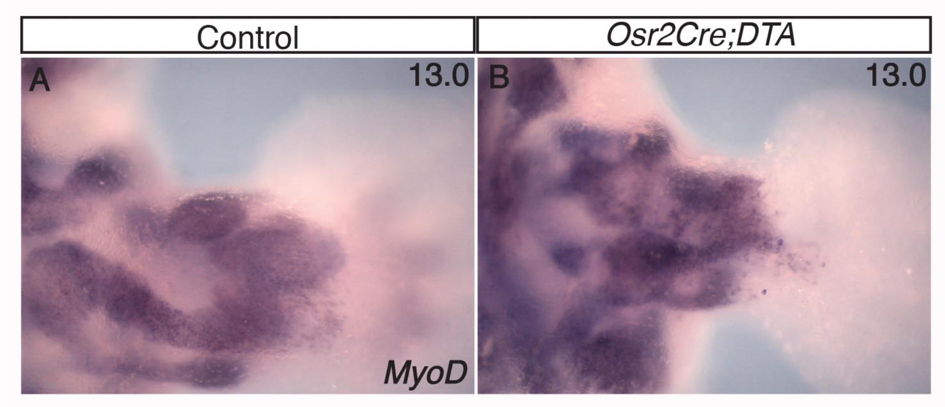

Figure S2. Muscle morphogenesis is disrupted in *ROSA26-eGFP-DTA;Osr2Cre* embryos. Related to Figures 2, 3 and 4
Dorsal view of control (A) and *ROSA26-eGFP-DTA;Osr2Cre* mutant (B) forelimbs at E13.0 processed by whole mount *in situ* hybridisation for *MyoD*. Nascent muscle bundles, aligned fibres and a central zeugopodal domain where *MyoD* expression is excluded can be seen in control (A). In mutant (B) MyoD-positive cells are present but are distributed in an abnormal pattern.

A

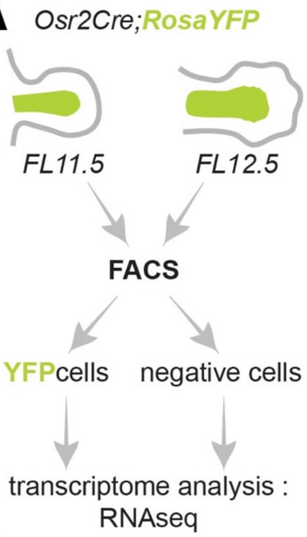

B

Gene	RC +/- 11.5	FC +/- 11.5	p value 11.5	RC +/- 12.5	FC +/- 12.5	p value 12.5
Keratocan	1943.42/58.07	27.06 up	0.0000129	5505.85/206.01	23.2 up	1.35E-04
Epiphycan	167.53/7.73	17.52 up	0.000089	204.05/35.25	7.58 up	0.021
Decorin	956.06/122.14	6.3 up	3.64E-05	6582.79/755.01	7.6 up	0.0037
Fibromodulin	623.25/89.05	5.7 up	6.42E-05	2724.94/633.71	3.7 up	0.001
Osteoglycin	1423.73/203.56	5.7 up	1.25E-04	4539.41/1679.31	2.4 up	0.008
Lumican	5720.96/970.32	4.8 up	6.33E-05	12496.12/2816.32	3.9 up	3.85E-04
Podocan	249.42/44.61	4.52 up	2.93E-04	318.21/124.2	2.2 up	0.0016
Osr2	2888.82/290.12	8.1 up	2.40E-05	1668.51/397.58	3.6 up	9.91E-05
Osr1	3706.34/1043.41	2.9 up	7.08E-05	4329.6/1900.15	2.0 up	0.002

Figure S3. SLRPs are enriched in the limb ICT/MCT population of cells. Related to Figure 6 and Supplementary Figure 4

(A) Schematic diagram of the strategy used to label and isolate ICT cells for transcriptome analysis using the combination of *Osr2Cre* deleter and *ROSAYFP* reporter with FACS to generate YFP+ and YFP- cell pools. (B) Table containing a selected list of some of the most differentially expressed genes comparing the transcriptome of YFP+ ICT/MCT cells with YFP- limb mesenchyme cells.

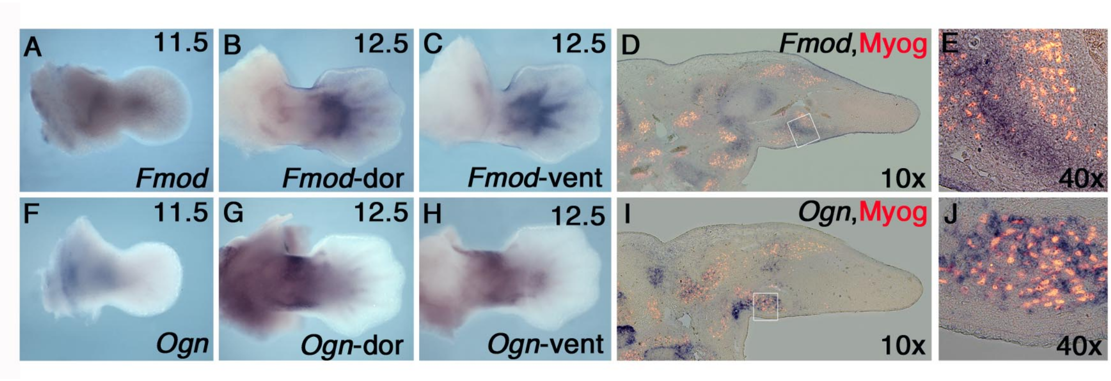

Figure S4. Expression profiles of SLRPs *Fmod* and *Ogn* in the forelimbs. Related to Figure 6. Dorsal view of wild type forelimbs at E11.5 (A,F) and E12.5 (B,G) and the ventral view (C,H) of the E12.5 limbs in B,G, processed for *Fmod* (A-C) and *Ogn* (F-H) by whole mount *in situ* hybridisation. Localisation of SLRP expression with muscle cells detected by section *in situ* hybridisation for *Fmo*d or *Ogn* followed by immunofluorescence for Myogenin at E12.5 and presented as 10X images (D,E). The region boxed in 10X images are 40X magnification (E,J).
