# Supplementary material for: Individual Limb Muscle Bundles Are Formed through Progressive Steps Orchestrated by Adjacent Connective Tissue Cells during Primary Myogenesis
Source: Cell Rep. 2020 Mar 10;30(10):3552–3565.e6. doi: 10.1016/j.celrep.2020.02.037 (PMC7068676; doi:10.1016/j.celrep.2020.02.037)
Supplement: Document S2. Article plus Supplemental Information [file mmc11.pdf]

Cell Reports

Article

Individual Limb Muscle Bundles Are Formed through Progressive Steps Orchestrated by Adjacent Connective Tissue Cells during Primary Myogenesis

Graphical Abstract

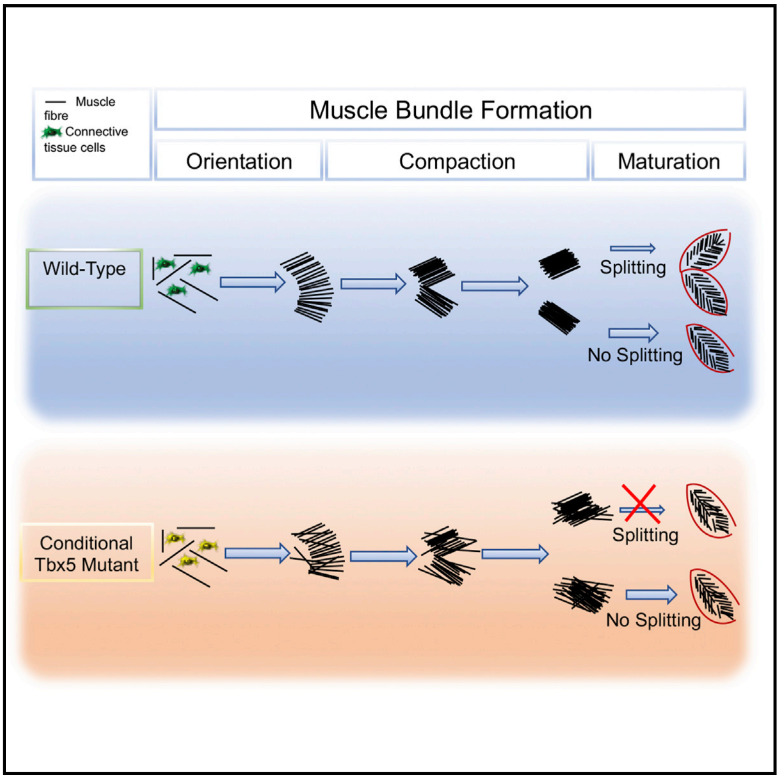

Highlights

- Characterization of the events that prefigure the formation of individual muscle bundles
- Direct demonstration of the role of connective tissue cells in muscle morphogenesis
- Identification of markers of limb irregular connective tissue (ICT)
- Demonstration of molecularly distinct ICT subdomains in the limb

Authors

Laurianne Besse, Caroline J. Sheeba, Mark Holt, ..., Donald Bell, Ania Kucharska, Malcolm P.O. Logan

Correspondence

malcolm.logan@kcl.ac.uk

In Brief

Besse et al. describe how differentiating muscle fibers are transformed into limb muscle bundles. Using gene deletion and genetic ablation, they demonstrate the importance of surrounding connective tissue cells in orchestrating muscle morphogenesis, identify markers of this population in limbs, and provide a model for how these cells influence muscle precursors.

Besse et al., 2020, Cell Reports *30*, 3552–3565
March 10, 2020 © 2020 The Author(s).
https://doi.org/10.1016/j.celrep.2020.02.037

CellPress

OPEN ACCESS
CellPress

Cell Reports
Article

Individual Limb Muscle Bundles Are Formed through Progressive Steps Orchestrated by Adjacent Connective Tissue Cells during Primary Myogenesis

Laurianne Besse,[1] Caroline J. Sheeba,[1] Mark Holt,[1] Maurice Labuhn,[1] Susan Wilde,[1] Eleanor Feneck,[1] Donald Bell,[2] Ania Kucharska,[3] and Malcolm P.O. Logan[1,4,*]
[1]Randall Centre for Cell and Molecular Biophysics, King's College London, Guy's Campus, London SE1 1UL, UK
[2]Light Microscopy, Francis Crick Institute, 1 Midland Road, London NW1 1AT, UK
[3]Stem Cell Biology and Developmental Genetics, Francis Crick Institute, 1 Midland Road, London NW1 1AT, UK
[4]Lead Contact
*Correspondence: malcolm.logan@kcl.ac.uk
https://doi.org/10.1016/j.celrep.2020.02.037

SUMMARY

Although the factors regulating muscle cell differentiation are well described, we know very little about how differentiating muscle fibers are organized into individual muscle tissue bundles. Disruption of these processes leads to muscle hypoplasia or dysplasia, and replicating these events is vital in tissue engineering approaches. We describe the progressive cellular events that orchestrate the formation of individual limb muscle bundles and directly demonstrate the role of the connective tissue cells that surround muscle precursors in controlling these events. We show how disruption of gene activity within or genetic ablation of connective tissue cells impacts muscle precursors causing disruption of muscle bundle formation and subsequent muscle dysplasia and hypoplasia. We identify several markers of the populations of connective tissue cells that surround muscle precursors and provide a model for how matrix-modifying proteoglycans secreted by these cells may influence muscle bundle formation by effects on the local extracellular matrix (ECM) environment.

INTRODUCTION

Three main tissues comprise the musculoskeletal unit: bones, muscles, and tendons. Unlike the bones and tendons of the limb, which are derived from the lateral plate mesoderm, the limb musculature is formed from muscle precursors that originate in the hypaxial domain of the somites adjacent to the limb and migrate into the limb bud periphery where they meet the resident precursors of the bones and tendons, to which they ultimately connect (Buckingham et al., 2003; Kardon, 2011). After muscle precursors have entered the limb bud, they undergo a rapid transformation to become organized into individual muscle bundles. Several studies in chicks have described the process of organizing dorsal and ventral pre-muscle masses into individual muscles in the leg (Kardon, 1998; Rodriguez-Guzman et al., 2007; Schroeter and Tosney, 1991a, 1991b). It is well established that muscle progenitors are autonomously programmed to undergo muscle differentiation; however, the signals that determine precisely what type of muscle they will form and, therefore, their shape and position in the limb are determined by environmental cues produced by cells of the limb bud into which these muscle precursors migrate (Chevallier et al., 1977; Grim and Wachtler, 1991; Spornitz, 1978; Kardon et al., 2002). More recently, in the developing limb buds, muscle connective tissue (MCT) has been shown to be an important source of such patterning signals to both nascent muscles and tendons (Hasson et al., 2010; Kardon et al., 2003; Mathew et al., 2011; Colasanto et al., 2016; Swinehart et al., 2013; Vallecillo-García et al., 2017). MCT is a sub-population of irregular connective tissue (ICT) and named on the basis that it surrounds and is embedded within nascent muscle tissue. MCT will ultimately contribute to muscle fascia. Little is known about the cellular and molecular mechanisms that control formation of the, in excess of, 40 individual muscles that are formed from the stream of muscle precursors that migrate into the limb. Although there is consensus on the importance of MCT for muscle morphogenesis, the mechanisms by which these cells affect muscle and tendon formation during embryonic development are not understood. This is compounded by the lack of obvious "pattern" in the distribution of MCT markers, such as *Tcf4*, that could serve as a template for where and when individual muscles eventually form and the paucity of alternative MCT/ICT markers.

Muscle morphogenesis normally occurs reproducibly and with high fidelity, as demonstrated by the mirror symmetrical array of muscles that are present in the left and right limbs of an individual and in the conserved patterns seen across different vertebrate species. However, minor variations in the number and placement of muscles can occur, with a classic example being an absence of the palmaris longus muscle in the flexor compartment of the forearm seen in approximately 15% of the population. More clinically significant, many congenital abnormalities have associated muscle hypoplasias/dysplasias. For example, the upper limb muscle defects present in Holt-Oram syndrome (HOS OMIM 142900), a dominant disorder associated with mutations in *TBX5* and characterized by upper limb and heart defects (Basson et al., 1997; Li et al., 1997) can be attributed, in part, to disruption of the MCT/ICT (Hasson et al., 2010). In the majority of cases, the reasons for muscle dysplasias or hypoplasias are

This is an open access article under the CC BY license (http://creativecommons.org/licenses/by/4.0/).

not understood and it is unclear if the root cause is due to failure to form a nascent muscle bundle or, alternatively, because forming muscle bundles subsequently degenerate.

A clear understanding of how muscle precursors behave to form individual muscle bundles is essential before analyzing the influence of the MCT/ICT in this process. By stage embryonic day 10.5 (E10.5), all the limb muscle precursors have entered the limb bud (Buckingham et al., 2003; Figure 1A). Over the course of 4 days, these cells differentiate into muscle fibers that are organized into discrete muscle bundles that are the templates of all the muscles of the adult limb (Figure 1; Delaurier et al., 2008). Thus, the events of primary myogenesis produce the mature pattern of individual limb muscles that enlarge and mature during subsequent events of secondary and post-natal myogenesis. There is no single marker described that can label all precursors throughout this time period. Therefore, to follow how individual muscle bundles are generated, we used a combination of *MyoD* (at E10.5) and myogenin (at E11.5 and later) to mark myoblasts and myocytes and myosin to mark the terminally differentiating myocytes and muscle fibers across a time course from E10.5 until E14.5. For simplicity of presentation, we focus on the dorsal zeugopodal muscles (extensor compartment of the forearm), although similar events were observed throughout the limb musculature (data not shown).

We describe a multistep process that leads to the formation of distinct muscle bundles. We show that cell orientation, clustering, compaction, and in some cases "splitting" or cleavage of nascent muscle bundles occur in an overlapping progression from a common pool of myocyte precursors before secondary myogenesis starts. Impairing the activity of MCT/ICT on the muscle precursors by deleting *Tbx5* from the MCT/ICT or by depleting these cells produces muscle and tendon patterning defects. We show that MCT/ICT is required for proper muscle morphogenesis, supporting previous findings (Hasson et al., 2010; Kardon, 1998; Mathew et al., 2011). We extend these findings and describe the muscle individuation steps that are controlled by the activity of MCT. By using tools to genetically label ICT/MCT and carrying out a transcriptome screen, we identify several members of the small leucine-rich repeat proteoglycan (SLRP) family as MCT/ICT markers and that each SLRP has a unique expression domain in limb ICT, indicating the existence of MCT/ICT subdomains. Furthermore, we show that the SLRP expression domains are disrupted in the mouse mutants following the targeted deletion of *Tbx5* in ICT/MCT and that this precedes the observed muscle-patterning defects. Together, our results support a model in which spatially distinct MCT/ICT territories organize cohorts of muscle precursors into muscle bundles.

RESULTS

Clustering, Orientation, and Fusion of Muscle Cells Are the First Cellular Events in Muscle Bundle Individuation

Between E10.5 and E12.5, the first wave of myoblast differentiation into post-mitotic, elongated myocytes that fuse to form multinucleated primary fibers occurs (Abmayr and Pavlath, 2012; Lee et al., 2013). Simultaneously, we detect the first evidence of these muscle precursors being organized, marked by a regular clustering and orientation of myogenin/myosin-positive myofibers (Figures 1A–1K). *MyoD*-positive myoblasts (Buckingham and Vincent, 2009; Murphy and Kardon, 2011) are initially grouped uniformly and with no obvious organization in a central territory (Figure 1A). An early event is the aggregation of cells into clusters resulting in the clearing of cells from a central domain (Figure 1C, arrow). This is further refined by E12 (Figures 1G and 1J) and E12.5 (Figures 1H and 1K). At E11.75, four prominent clusters are visible (Figure 1C): proximal (Pr) and posterior (Po) clusters that contribute to the upper arm (stylopod) muscles, e.g. biceps, brachialis, and triceps; and anterior (An) and distal (Di) clusters that contribute to the forearm extensor muscles (Figures 1C, 1F, and 1G–1L). At E11.75, multinucleated cells are detected, marking the beginning of myocyte fusion to form myofibers (Figure 1F, arrow); this process continues and by E12.5 the majority of cells are visible as fused myofibers (Figure 1K). At E12.5, the primary fibers have become aligned with one another along common orientation planes that prefigure the position of future muscle bundles (Figures 1K–1M), for example the extensor carpi radialis (ECR) in the anterior, the extensor digitorum communis (EDC) centrally, and the extensor digitorum lateralis (EDL) in the posterior. By E13, myofibers are organized into highly ordered and compacted units (Figure 1L), some of which divide further so by E14.5 all the individual muscles present in the adult can be distinguished (Figure 1M; Delaurier et al., 2008). This analysis reveals that prior to the formation of individual muscle bundles, nascent fibers are organized along distinct orientation planes and that these orientation planes prefigure the positions of muscles.

Quantification of Muscle Fiber Orientation

To analyze the emergence of these distinct myofiber units in the dorsal zeugopod (Figures 2A–2D, boxed region) between E11.5 and E12.5, we labeled early and late myocytes and nascent fibers by using a combination of RNA *in situ* hybridization and immunohistochemistry to detect myogenin and myosin and used confocal microscopy to scan the result of immunostainings in dorsal forelimbs (Videos S1, S2, S3, and S4). To quantify the process of fiber orientation and see the emergence of distinct myofiber clusters with common orientation planes, cells expressing both myogenin and myosin were manually outlined on every optical section of a z stack (Videos S1, S2, S3, and S4). Projections of these binary files representing each Z plane were then produced (Figures 2E–2H; see STAR Methods). These projections reveal that at E11.5 the orientation of early myocytes is not completely random. At E11.75 the An and Di clusters are becoming more distinguished and cells within each cluster become progressively more precisely aligned and compacted. By E12.5, distinct fiber clusters are identifiable both deep and more superficial. The two deeper clusters are aligned perpendicular to the other myofibers (Figure 2H, asterisks; Video S4).

To further study the processes of cell orientation and clustering and their progressive refinement, we developed a method to color-code the outlined cells based on their similar orientation angles. Using the statistical method Central Moments (see STAR Methods), we derived orientation values (effectively 1–180°) and assigned different colors to value ranges. This enabled us to identify sub-groups of fibers within the limb based on their similar

OPEN ACCESS
CellPress

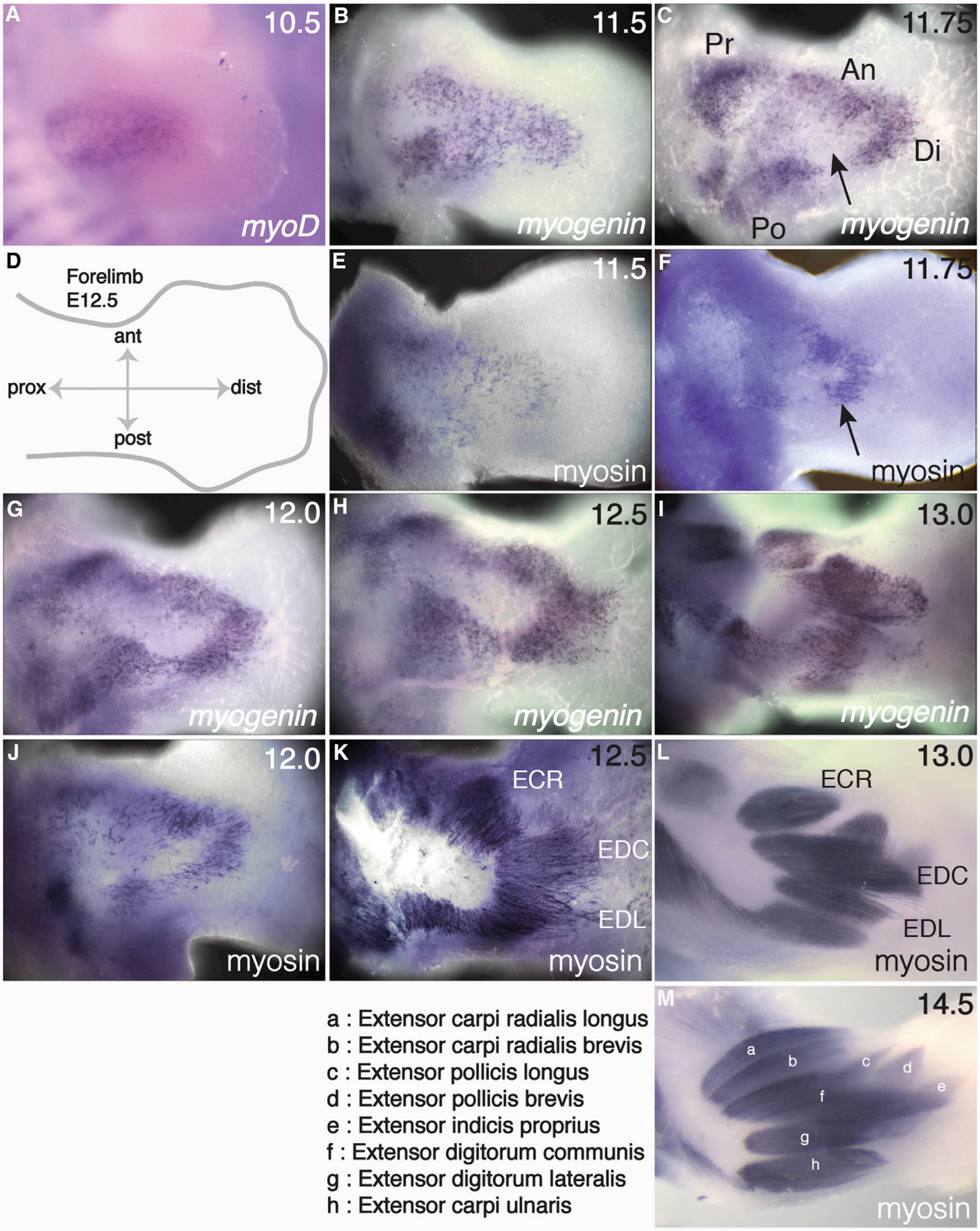

Figure 1. Individual Muscle Bundles Form through Progressive Series of Steps

Dorsal view of control forelimbs from E10.5 (A) to E14.5 (M) embryos. Muscle progenitors are detected by *in situ* hybridization for *MyoD* (A) or *Myogenin* (B, C, G, H, and I) and immunohistochemistry to detect Myosin (E, F, J, K, L, and M). The limbs are oriented from proximal to distal as shown in the schematic E12.5 forelimb (D). Arrow in (C) shows an area devoid of myogenin positive cells. Arrow in (F) represents the beginning of myocyte fusion. Individual muscles are identified and listed in (M).

orientation (Figure 2H′). This method transforms the monochrome projections into multi-color images that illustrate the sequential increase in fiber organization (Figures 2E′–2H′). At E11.5, the majority of cells are orientated non-randomly (anisotropic) diagonally to the Pr-Di axis of the limb (from left to right in Figure 2). Significantly, this demonstrates that, even early in the pathway toward muscle bundle formation, myocytes have some degree of orientation before the formation of muscle fibers.

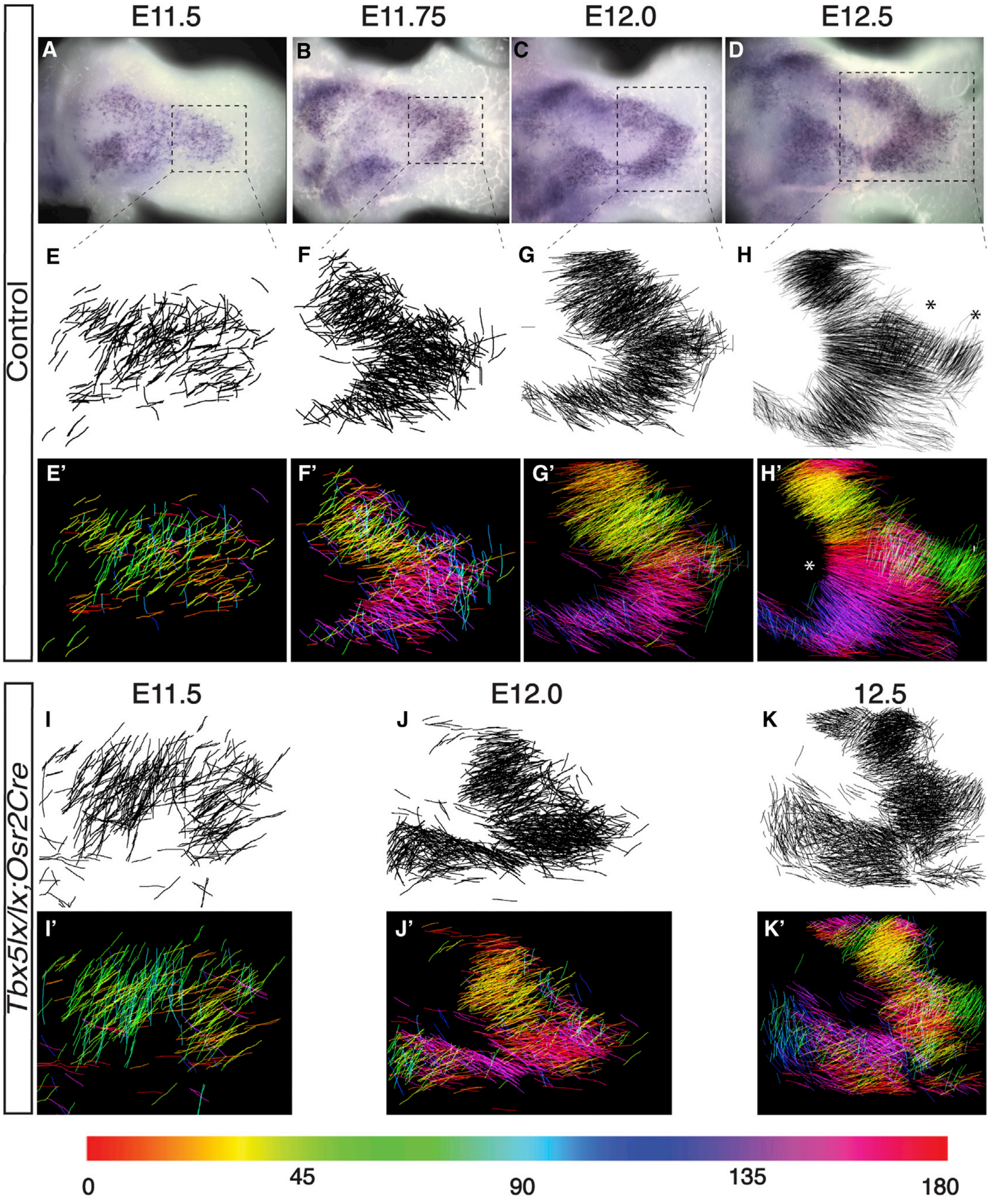

Figure 2. Orientation, Clustering, and Compaction of Nascent Muscle Fibers Prefigure Muscle Bundle Formation and Are Disrupted Following Conditional Deletion of *Tbx5*

(A–D) Dorsal view of E11.5 (A), E11.75 (B), E12.0 (C), and E12.5 (D) control forelimbs with muscle cells detected by *Myogenin in situ* hybridization to illustrate the region analyzed by immunohistochemistry in (E)–(K). Dotted line squares in (A)–(D) show the approximate area where cell vectors shown in (E)–(H) have been drawn.

(E–K) Projection of cell vectors drawn from a Z series of confocal scans of limbs at the stages indicated, stained by whole mount immunohistochemistry for myogenin and myosin in control E11.5 (E), E11.75 (F), E12.0 (G), E12.5 (H), and *Tbx5*$^{lx/lx}$*;Osr2Cre* mutant E11.5 (I), E12.0 (J), and E12.5 (K). The vector lines outline cell orientation.

(E'–K') Projection of the color-coded cell vectors for control (E'–H') and mutant (I'–K') forelimbs. Each cell vector has been assigned a color value corresponding to a range of angle values from 0° to 180°, shown on the rainbow ruler.

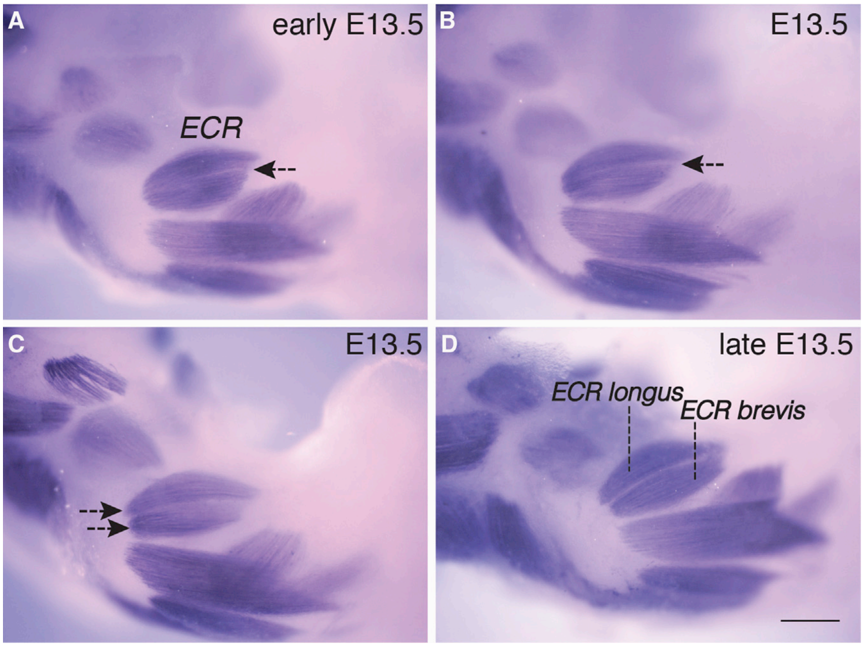

Figure 3. Cleavage of Muscle Bundle Is a Step in the Morphogenesis of Only Some Muscles

Consecutively staged dorsal views of four E13.5 forelimb zeugopods indicate the progressive cleavage of the single extensor carpi radialis (ECR) bundle into two distinct ECR longus and ECR brevis muscles. Muscles (blue/purple) are stained by whole-mount immunohistochemistry to detect Myosin.

(A) In the most immature specimen, cleavage of the single extensor carpi radialis (ECR) bundle has started at the distal end (black arrow).

(B) Cleavage extends from distal to proximal and is almost complete.

(C) Separation of the single bundle into two discrete units is complete at the proximal end of the bundles (black arrows).

(D) At the end of the cleavage event, two distinct ECR longus and ECR brevis bundles are formed. Scale bar, 200 μm.

By E11.75, cellular rearrangements lead to the formation of An and Di clusters with distinct orientation vectors (Figures 2B and 2F, labeled yellow and magenta in 2F′). A distal domain is also emerging with fibers aligning vertically. During subsequent steps, up to E12.5, fibers become aligned along a common proximal boundary (Figure 2H′, asterisk) but cluster into nascent bundles with their own distinct orientation vectors (labeled yellow, red, magenta, and blue in Figure 2H′). The distal domain becomes organized into two distinct clusters (labeled green Figure 2H′) that prefigure the extensor pollicis longus (EPl), extensor pollicis brevis (EPb), and extensor indicis proprius muscles (Figure 1M). A key organization event, therefore, is the alignment of groups of fibers along particular orientation vectors by E12.5, which prefigures where muscle bundles form. This process may enable and/or facilitate these distinct myofiber clusters to undergo the compaction into defined muscle bundles that occur at E13.0 (Figure 1L). In summary, these results show that by E12.5–E13, extensive muscle fiber organization that prefigures individual muscle bundles is achieved by an overlapping series of orientation and clustering of muscle precursors, as they differentiate and fuse, rather than by what has previously been described as muscle splitting, which implies the subdivision of a coherent, larger domain of cells into smaller groups of cells.

A Minority of Muscles Are Formed through Cleavage of Visually Distinct Bundles of Muscle Fibers

Some forming muscles undergo a further refinement step that involves the cleavage of existing, coherent muscle bundles to form two smaller muscle bundles, a process that resembles a muscle splitting event. By E13, the ECR precursor bundle is distinct (Figures 1L and 3A). Over the course of approximately 6–12 h, this bundle divides in two. This starts at the distal end (Figures 3A and 3B, arrows) and progresses proximally (Figures 3C and 3D, arrows). A similar event (not shown) results in the cleavage of a single EP precursor to form the EPl and EPb, which are distinct by E14.5 (Figure 1M). In the dorsal zeugopod, this mechanism of muscle bundle individuation is limited to longus and brevis muscles that are closely associated with one another and share similar origins and insertions.

Osr2IRESCre Is Expressed in ICT, Including MCT

In a previous study using a pan-limb mesenchyme, tamoxifen-inducible cre, we demonstrated, indirectly, that *Tbx5* acts in MCT to regulate muscle (and tendon) morphogenesis (Hasson et al., 2010; Gao et al., 2011). To directly assess the function of *Tbx5* in MCT, we sought to identify a cre deleter line that would enable us to target MCT specifically and genetically label this population of cells.

The *odd-skipped* related transcription factors *Osr1* and *Osr2* are expressed in ICTs, including the MCT in chick and mouse limbs (Stricker et al., 2006; Vallecillo-García et al., 2017). We used the *Osr2IRESCre* allele (Lan et al., 2007) (hereafter referred to as *Osr2Cre*) to target gene deletion and marker gene activation in the limb ICT, including extensive areas of the MCT. The *Osr2Cre* produces cre activity in the zeugopod (forearm) region and is less extensive in more proximal (stylopod and girdle) domains of the forelimb equivalent to that previously reported (Figures S1A and S1B). Figures S1 *Osr2Cre* activity (reported by GFP expression from the cre-inducible reporter) is excluded from Sox9-expressing cartilage precursors, with the exception of a population of joint interzone cells between the humerus and ulna (Figures S1C and S1D), consistent with a previous report (Gao et al., 2011). This exclusion of staining is confirmed when dissociated limb bud cells are stained in culture (Figure S1E). Distinct GFP-expressing or Sox9-expressing cells are detected. Cre activity is observed in dorsal and ventral domains surrounding and embedded within, but not overlapping with, MyoD-positive muscle precursors at E10.5 (data not shown), E11.5, and E12.5 (Figures S1F and S1G). This non-overlapping patterning of GFP and MyoD staining was also confirmed when dissociated limb bud cells were stained in culture. Significantly,

Osr2Cre activity is detected in Tcf4-positive MCT cells associated with the dorsal and ventral muscle masses (Figures S1I and S1J). In dissociated limb bud cells in culture, expression of Tcf4 is observed in GFP-expressing cells (Figure S1K). However, the domain of *Osr2Cre* activity is broader (Figure S1I). Thus, *Osr2Cre* targets a wider population of MCT and ICT cells than that labeled by *Tcf4*. *Tcf4* is also expressed in the distal cartilage precursors (Figure S1I). We observe *Osr2Cre*-targeted cells surrounding and interspersed between nascent muscle bundles at E13.5 (Video S5). In summary, during stages encompassing primary myogenesis, *Osr2Cre* targets a population of ICT cells, including a large portion of MCT that surrounds the limb muscle precursors but is excluded from the muscle and the great majority of cartilage precursors.

Deletion of *Tbx5* by *Osr2Cre* Produces Muscle and Tendon Patterning Defects

To directly test the activity of *Tbx5* in MCT and demonstrate the efficacy of the *Osr2Cre* deleter to target this cell population, we used the *Osr2Cre* line to conditionally delete *Tbx5* in a large proportion of limb MCT progenitor cells from E10.5. *Tbx5lx/+;Osr2cre* heterozygous embryos have no apparent phenotype (Figures 4A, 4C, 4E, 4G, 4I, 4K, 4M, and 4O). In contrast, *Tbx5lx/lx;Osr2Cre* homozygous conditional mutant embryos (hereafter referred to as mutants) have defects in muscle morphogenesis (Figures 4B, 4D, 4F, 4H, 4J, 4L, 4N, and 4P). We used immunofluorescence to examine the morphology and location of muscles and associated tendons and focused on the dorsal forearm (zeugopod) muscles in the forelimb. We consistently detect 4 distinct abnormalities in mutant limbs: (1) failure of muscle bundles to divide to form two distinct muscles, a failure of cleavage or splitting; (2) formation of smaller, hypoplastic muscles; (3) absence of a muscle bundle; (4) a larger muscle bundle; and (5) stray, misaligned fibers (Figure 4). The ECR longus (ECRl) and ECR brevis (ECRb) are two adjacent muscles in the radial, posterior compartment of the forearm (Figures 1M, 3, 4G, 4M, and 4O). Instead, a single muscle body with a single tendon attachment is found in the equivalent location in the mutant (Figures 4H, 4L (arrowed), 4N, and 4P). Similarly, the EPl and EPb do not form and a single muscle and tendon is present (Figures 4M and 4N). The EDC muscle lies superficial and central in the forearm (Figure 4E, outlined with dashed line). In the mutant, this muscle is smaller and lacks the distinctive fusiform shape (Figure 4F, outlined with dashed line), although it attaches to digits 2–5, consistent with the EDC muscle (Figures 4E and 4F). The EDL muscle lies just below the EDC (Figures 4E, 4I, and 4K, asterisk). This muscle is absent in the mutant (Figures 4F, 4J, and 4L, asterisk). The extensor carpi ulnaris (ECU) lies posterior to the EDL and has an associated tendon that extends to the 5th metacarpal (Figure 4I, arrowhead). In the mutant, this muscle is larger and the single associated tendon splits to insert in digit 5 and 4 (Figures 4J and 4L, arrowhead). One striking observation in mutant limbs at E13.5 is the presence of stray, misaligned fibers (Figure 4D, arrow), suggesting a degree of disorganization of some nascent fibers as they begin to aggregate into clusters. These misaligned fibers appear transient because they are not detected at E14.5 (Figure 4H).

Deletion of *Tbx5* by *Osr2Cre* Disrupts Clustering and Aggregation of Muscle Precursors

To study the origin of the soft tissue abnormalities in the mutant, we stained limbs at earlier stages by using markers of muscle precursors. Analysis of Pax3 at E10.5 and MyoD and myogenin at E11.5 showed no differences between the mutant and control limbs (Figures 5A–5F), but abnormalities can be seen at E12.0 and become more pronounced at E12.5 (Figures 5G–5J). Notably, some muscle precursors fail to clear from a central domain of the dorsal forelimbs (Figure 5G–5J and asterisk in 5H). Muscle precursors are more diffuse in the mutant, and some cells fail to undergo the same degree of clustering and compaction that help segregate cohorts of muscle precursors. For example, in the posterior of the limb, these processes lead to the separation of two domains of muscle precursors (arrowheads in Figures 5G and 5I), and this fails to occur at equivalent stages in the mutant (arrowhead, Figures 5H and 5I). This abnormal distribution of muscle precursors and fibers is reproducible, suggesting that the abnormal clustering and compaction consistently observed at early stages is responsible for the absent and abnormally shaped muscles that result later (Figure 4).

Disruption of fiber orientation and compaction was apparent by applying our Central Moment analysis method to mutant limbs stained for myogenin and myosin (Figures 2I–2K′; Videos S6, S7,and S8) and by comparing these to control samples described earlier (Figures 2A–2H′). Disruption in the extent of orientation is clearly detectable at E12.0 (Figures 2J and 2J′ compared to Figures 2G and 2G′) and E12.5 (Figures 2K and 2K′ compared to Figures 2H and 2H′) in the mutant with stray fibers present. Although similar orientation planes of muscle fibers are observed in the mutant, these cells are less ordered and less compacted than control samples.

To compare the effect of disrupting *Tbx5* activity in ICT cells to the effect of depleting ICT cells, we took advantage of the ROSA26-GFP-DTA (Ivanova et al., 2005) to achieve genetic ablation of ICT by cre-mediated expression of the diphtheria toxin (DTA). *ROSA26-eGFP-DTA;Osr2Cre* embryos were not viable beyond E13.0, which restricted analysis up to this time point. Analysis of *ROSA26-eGFP-DTA;Osr2Cre* limbs at E13.0 showed dramatic disorganization of muscle precursors, more severely than that observed following deletion of *Tbx5* in the ICT (compare Figure S2 with Figures 4C, 4D, and 5I–5J), suggesting that deletion of *Tbx5* perturbs only some aspects of the function of ICT in muscle morphogenesis and that in the absence of ICT muscle development is adversely affected.

Despite their abnormal shape and location within the limb, muscles in the *Tbx5lx/lx;Osr2Cre* homozygous conditional mutant do become innervated and can control movement of the limb skeleton (Video S9). Mutant pups at post-natal day 0 (P0) appear to have difficulty fully pronating the forelimb to plant the ventral surface of the paw on the surface and instead the paw is held in a more supine position, often leading to the pup walking on the edge or back (dorsal surface) of the paw. These results definitively demonstrate that *Tbx5* acting within the MCT/ICT has an important influence on the morphogenetic processes that produce individual muscle bundles and that in the absence of *Tbx5* muscle differentiation and aspects of secondary myogenesis, such as muscle growth and innervation, can occur.

OPEN ACCESS
CellPress

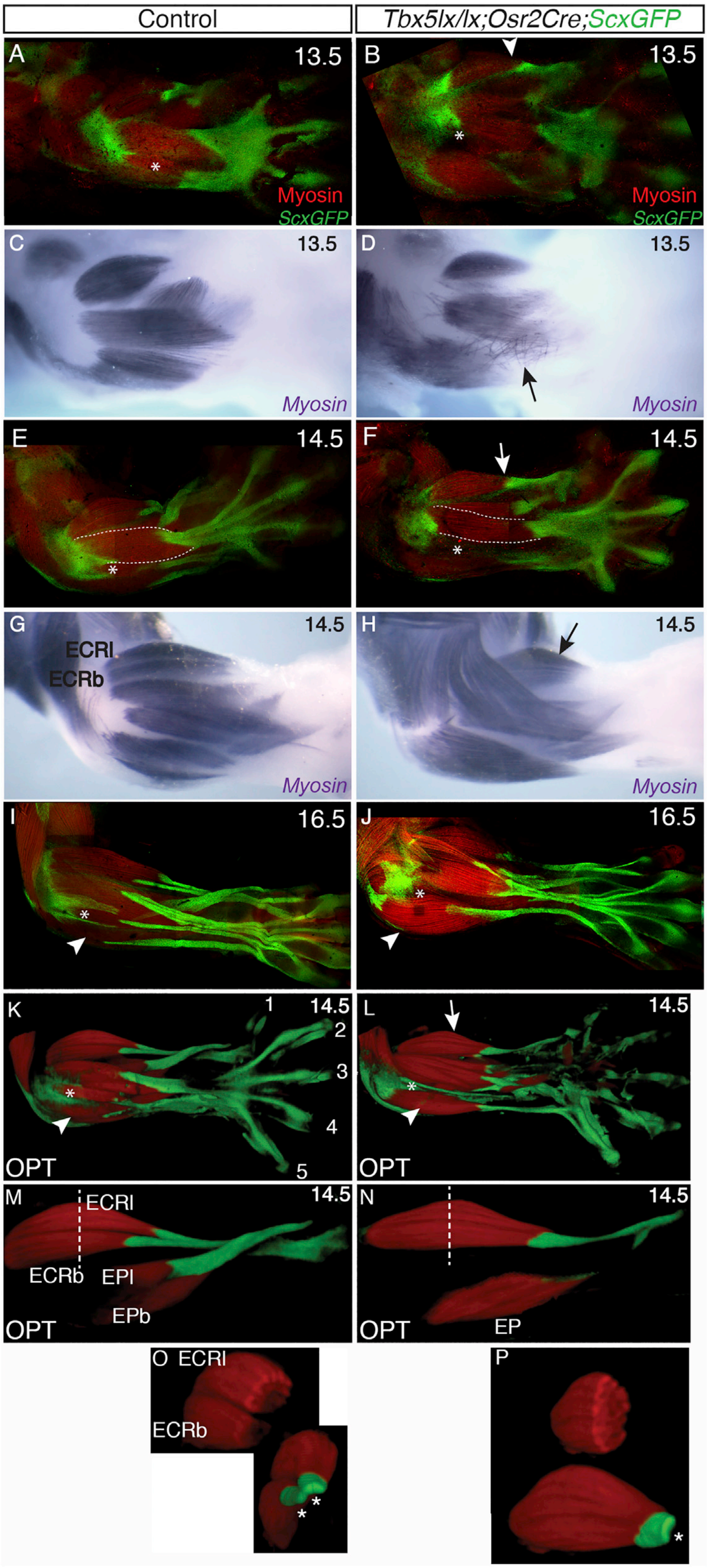

Figure 4. Deletion of *Tbx5* by *Osr2Cre* Produces Muscle and Tendon Patterning Defects

Muscles and tendons labeled for myosin and *ScxGFP*, respectively. Dorsal view of control E13.5 (A and C), E14.5 (E, G, K, M, and O), and E16.5 (I) forelimbs and equivalent stages of *mutant* E13.5 (B and D), E14.5 (F, H, L, N, and P), and E16.5 (J) forelimbs. Black and white arrows indicate single ECR bundle (H) and a single tendon (B, F, and L) in mutant. Optical projection tomography (OPT) scans of control (K) and mutant (L) limbs at E14.5. Optical dissection of the ECRl, ECRb, EPl, and EPb of the sample shown in (K) (M). Equivalent optical dissection of the mutant shown in (L) showing single ECR and EP bundles (N). Dotted white lines in (M) and (N) show approximate position of optical slice through the 3D reconstruction made to generate the top images in (O) and (P), respectively. Bottom panels in (O) and (P) show rotated views of ECR to show the 2 tendons of ECRl and ECRb in the control and the single ECR tendon in the mutant (asterisks).

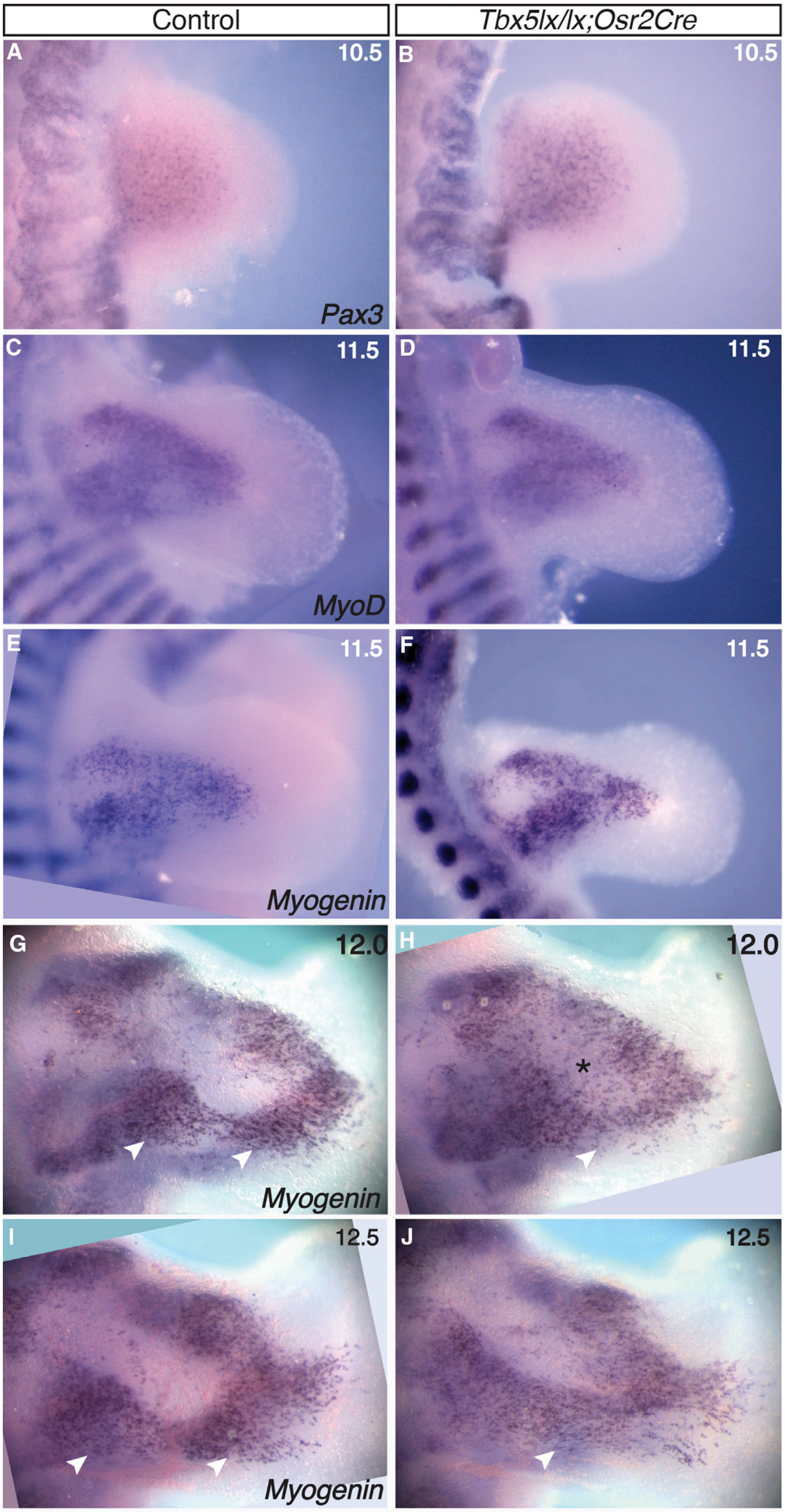

Figure 5. Muscle Patterning Defects in *Tbx5^lx/lx^;Osr2Cre* Mutants Are Detected at E12.0

Dorsal view of control (A, C, E, G, and I) and mutant (B, D, F, H, and J) forelimbs at E10.5 (A and B), E11.5 (C–F), E12.0 (G and H), and E12.5 (I and J). Muscle cells are detected by *in situ* hybridization against *Pax3* (A and B), *MyoD* (C and D), or *Myogenin* (E, F, G, H, I, and J). Arrowheads and asterisks point to clustering defects in the mutant forelimbs (J) compared to the controls (I).

OPEN
ACCESS
CellPress

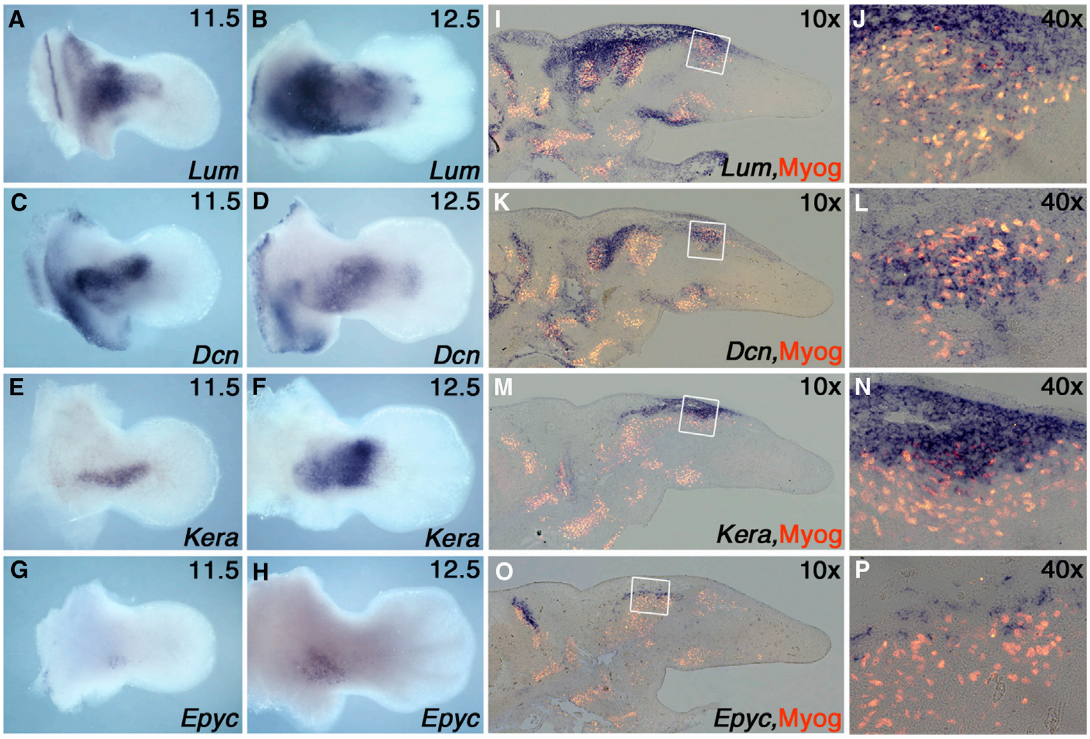

Figure 6. Expression Profiles of SLRP Genes in the Forelimbs Identify ICT Subdomains

Dorsal view of wild-type forelimbs at E11.5 (A, C, E, and G) and E12.5 (B, D, F, and H) processed with probes for *Lum* (A and B), *Dcn* (C and D), *Kera* (E and F), and *Epyc* (G and H) by whole mount *in situ* hybridization. Co-localization of SLRP expression with muscle cells were detected by section *in situ* hybridization followed by immunofluorescence for Myogenin at E12.5 (I–P) and shown at 10× magnification (I, K, M, and O). The region boxed in 10× panels is shown at 40× magnification (J, L, N, and P).

SLRP Proteins Are Enriched in MCT/ICT Progenitors and Identify Distinct ICT Subdomains

The lack of ICT markers has limited progress in understanding the functions of this tissue. To tackle this problem, we carried out a transcriptome screen to identify markers of the ICT that may also be important in ICT function. We combined the *Osr2Cre* allele with the *ROSAYFP* reporter transgenic to render the ICT cells GFP positive and then used fluorescence-activated cell sorting (FACS) to isolate the GFP-positive and -negative populations from limb buds at E11.5 and E12.5 (Figure S3A; STAR Methods). We then compared the transcriptome of these cell populations using RNA sequencing (RNA-seq). This screen successfully identified many new markers of ICT, and gene lists were particularly enriched for members of the SLRP family (Figure S3B; data not shown). SLRPs are ECM molecules that can bind various types of collagens, cytokines including transforming growth factor β (TGF-β), and several signaling receptors and regulate collagen fibrillogenesis, fibril organization, and matrix assembly, as well as cell proliferation, adhesion, migration, and differentiation (Merline et al., 2009). We confirmed the expression of *Keratocan* (*Kera*), *Decorin* (*Dcn*), *Lumican* (*Lum*), *Epiphycan* (*Epyc*), *Fibromodulin* (*Fmod*), and *Osteoglycin* (*Ogn*) by RNA *in situ* hybridization in whole-mount limb buds at E11.5 and E12.5 and on E12.5 sections (Figures 6 and S4). We chose to focus on stages E11.5 to E12.5, as our previous genetic studies demonstrated this to be a critical window of *Tbx5* activity in the ICT (Hasson et al., 2010) and this is the time frame when the first indication of muscle bundle individuation becomes apparent (Figure 1). Each SLRP has a unique expression pattern and is not expressed uniformly throughout the ICT but instead in subdomains of the limb ICT (Figure 6; Figure S4), which is more obvious when analyzed in sections (Figures 6I–6P; Figures S4D, S4E, S4I and S4J). *Lum* has a broader expression domain, predominantly because of its proximal limb expression, where it is either exclusively expressed (Figure 6I) or overlaps with *Dcn, Kera*, and *Epyc*. In addition, *Lum* is also expressed in the zeugopodal domain along with the largely zeugopodally expressed *Dcn*, *Kera*, and *Ogn* (Figure 6; Figure S4). SLRP subdomains can also be largely non-overlapping, for example *Dcn* and *Kera* (Figures 6K and 6M). *Fmod* has a distal expression domain compared to other tested SLRPs, and it is also expressed in the ectoderm (Figures S4A–S4D). To co-localize ICT and muscle precursors, in the same section, RNA *in situ* hybridization on sections for SLRP transcripts was followed by immunohistochemistry for myogenin. *Lum*, *Dcn*, and *Ogn* are expressed in ICT surrounding and embedded within some, but not all, nascent muscle bundles. *Ker*, *Epyc*, and *Fmod* are more restricted to the ICT surrounding nascent bundles and are less obvious within coalescing groups of muscle precursors.

We also analyzed the expression pattern of selected SLRPs in the forelimbs of *Tbx5lx/lx;Osr2Cre* homozygous conditional mutant embryos to establish if deletion of *Tbx5* had any effect

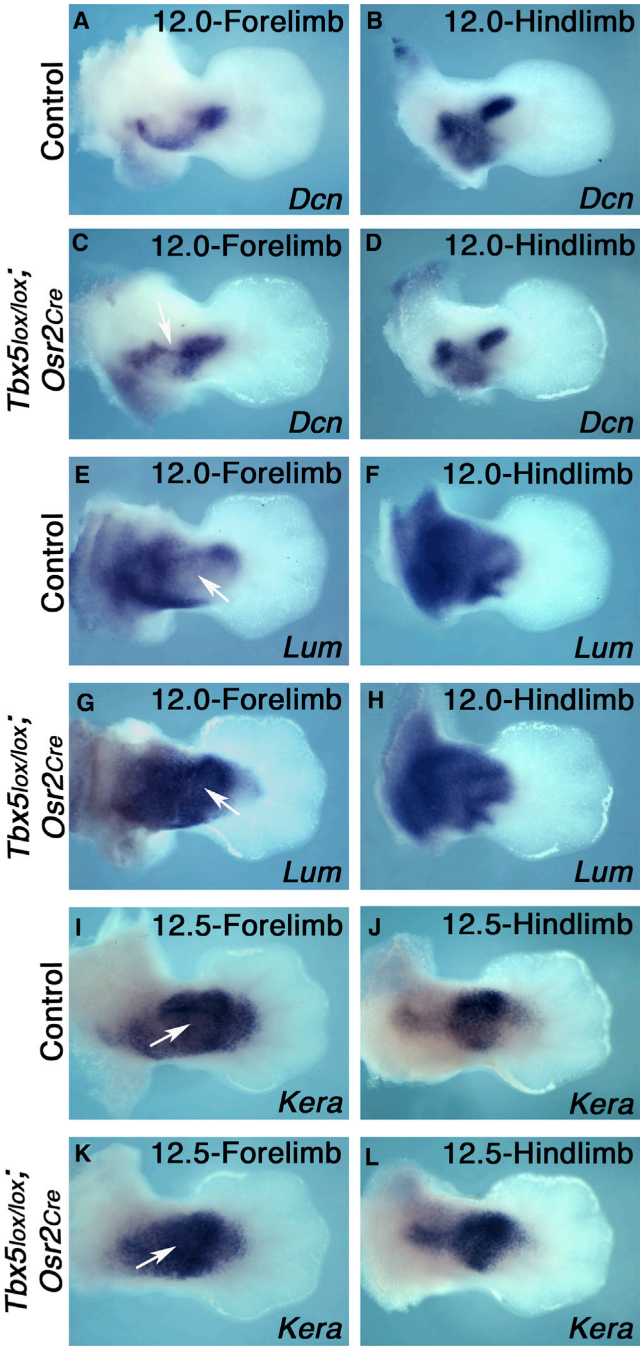

Figure 7. SLRP Expression Domains Are Altered in *Tbx5*$^{lx/lx}$*;Osr2Cre* Mutants

Dorsal view of wild-type forelimbs (A, E, and I) and hindlimbs (B, F, and J), and mutant forelimbs (C, G, and K) and hindlimbs (D, H, and L) between E12 to E12.5 processed for *Dcn* (A–D), *Lum* (E–H), and *Kera* (I–L) whole mount *in situ* hybridization. Arrow and arrowhead indicates the ectopic domain of *Dcn* and absence of its expression in the posterior zeugopodal region in mutant, respectively (C). Arrows in (E) and (I) show the central zeugopodal domain where *Lum* and *Kera* are generally excluded or expressed at low levels and arrows in (G) and (K) show the ectopic expression of these SLRPs in the central domain.

on their expression domains (Figure 7). We processed the cognate hindlimb of each control (*Tbx5lx/+;Osr2Cre* heterozygous) and mutant forelimb to serve as an internal staging control. Although the expressions of the SLRPs analyzed were consistent across hindlimb samples, alteration in the expression domains of *Dcn*, *Lum*, and *Kera* were detected in mutant forelimbs compared to controls (Figure 7). A restricted, ectopic domain of *Dcn* is observed (Figure 7C, arrow), whereas one of the normal domains was absent (Figure 7C, arrow). *Lum* and *Kera*, which are both normally excluded from a central zeugopodal domain (Figure 7E, 7I, arrow), are ectopically expressed in this region in the mutants (Figures 7G and 7K). Together, these results demonstrate that SLRP expression domains are disrupted following deletion of *Tbx5* in ICT.

DISCUSSION

We define the dynamic course of cellular events that lead to the formation of distinct limb muscle bundles. These include a progressive series of cell orientation, clustering, and compaction to form muscle bundles that, in some cases, undergo a further cleavage step. We demonstrate that the majority of limb muscles are formed through a process of orientation of precursor muscle myofibers, which prefigures subsequent clustering and compaction to form individual muscle bundles. These events occur as myofibers are undergoing terminal differentiation. This observation of orientation of muscle fibers prior to muscle bundle formation is consistent with studies of the chick hindlimb (Kardon, 1998; Schroeter and Tosney, 1991a, 1991b). Our Central Moments analysis extends these observations and describes the dynamic process of fiber orientation in which collections of fibers initially orientated in many directions (more isotropic) become organized into groups with parallel fibers in the same direction (increasingly anisotropic). Significantly, our analysis shows that initially large domains of fibers with similar orientation planes are present that encompass the precursors of multiple, individual, future muscle bundles. These larger domains of fibers are progressively refined into smaller cohorts of fibers with common orientation planes that prefigure individual muscle bundles or pre-muscle bundles that subsequently undergo cleavage. The process of forming individual muscle bundles is often referred to as muscle splitting or cleavage (Schroeter and Tosney, 1991a, 1991b), and recent studies have implicated vascular endothelial growth factor (VEGF) signaling from endothelial cells to be critical for this process (Tozer et al., 2007). Here, we show that the event that can be considered as muscle splitting (the cleavage of a single, coherent aggregate of muscle fibers into two smaller parts) is a later refinement event that contributes to the individuation of a minority of limb muscles. We show a specific example of cleavage from a single parental bundle in the dorsal/extensor compartment of the zeugopod that generates the ECRl and ECRb muscles. The earliest phases of muscle bundle formation occur in a consistent pattern, suggesting the series of events controlling this process are tightly regulated and are responsible for the regular array of muscle bundles that are formed by the end of primary myogenesis (around E14.5). Later events of myogenesis enlarge and refine the mature pattern of individual muscle bundles.

By deleting *Tbx5* specifically in the ICT/MCT, we show that partial disruption of the cellular events we describe that lead to muscle individuation has predictable and reproducible effects on the final size and shape of muscle bundles and can, in some cases, lead to the absence of muscle bundles. A role for MCT in limb soft tissue morphogenesis has been shown previously (Hasson et al., 2010; Kardon et al., 2003). More recently, recent studies have proposed molecular mechanisms for how MCT may act through both ECM and signaling pathways, such as Cxcl12/Cxcr4, to affect muscle precursor migration and proliferation (Vallecillo-García et al., 2017). Here, we identify some of the early cellular events regulated by the MCT that control muscle morphogenesis and that can become disrupted in the absence of *Tbx5* activity. Correct orientation of muscle fibers is disrupted in *Tbx5* mutants with compromised MCT/ICT function, indicating the importance of MCT/ICT for this early step in muscle morphogenesis. Because orientation of muscle fibers precedes subsequent steps that further refine individual muscle bundle formation (our results; Kardon, 1998), disoriented fibers in the mutants would be predicted to contribute to the observed muscle patterning defects. Disruption of fiber orientation was more severely disrupted following genetic ablation of ICT, corroborating the coordination between the muscle cells and the surrounding connective tissue for the earliest steps of muscle patterning (Hasson et al., 2010; Kardon et al., 2003; Mathew et al., 2011; Murphy et al., 2011). Our results indicate that at least some aspects of the muscle hypoplasia/dysplasia seen in HOS likely arise from a disruption of some of the earliest events of muscle morphogenesis and that absence of muscle likely occurs from a failure to form the primordia of the muscle bundles rather than a subsequent degeneration of a formed muscle bundle.

The presentation of muscle patterning defects following deletion of *Tbx5* in ICT is not identical to what we observed using a tamoxifen-inducible, pan-mesenchymal cre deleter, *Prx1CreERt2* (Hasson et al., 2010), although these original mutants were not analyzed to the same level of detail as the mutants described here. In our analysis of the *Tbx5lx/lx;Osr2Cre* homozygous conditional mutant, we have identified an absence of muscles and a failure of muscle bundle cleavage, whereas extra muscle bundles were observed in the *Tbx5lx/lx;Prx1CreERt2* mutants. The differences in phenotype can most likely be explained by technical differences between the two approaches. The events of muscle patterning, which are controlled, in part, through the activity of *Tbx5* in ICT/MCT, occur during a relatively narrow time window, around E11.5–12.5. Because there are variations in the timing and extent of cre activity following tamoxifen administration in each embryo, this factor could significantly impact phenotypes given the narrow time frame when *Tbx5* is required for muscle patterning. Consistent with this idea, variation in muscle mispatterning phenotypes were observed in *Tbx5lx/lx;Prx1CreERt2* mutants (Hasson et al., 2010). In marked contrast, use of conventional cre with the Osr2Cre line produced reproducible muscle mispatterning phenotypes, indicating that deletion of *Tbx5* within ICT at a fixed time disrupts normal events of muscle patterning with consistent, predictable outcomes.

We have identified members of a class of molecules, the SLRPs, which serve as novel markers of ICT/MCT and are candidates to have roles in ICT/MCT control of soft tissue morphogenesis. SLRPs are matricellular proteins that have well-established roles in modulating matrix assembly (Chen and Birk, 2013). Several lines of evidence suggest a role for SLRPs in muscle formation, repair, and disease. SLRPs can affect several signaling pathways implicated in muscle, for example myostatin, TGF-β, and insulin growth factor (IGF) (Lee et al., 2016; Schaefer and Iozzo, 2008; Zhou et al., 2011). Expression patterns of SLRPs are altered in Duchenne muscular dystrophy (DMD) and muscle injury (Casar et al., 2004; Fadic et al., 2006; Zanotti et al., 2005), and Dcn and Fmod have been implicated in regulating myogenesis (Brandan et al., 2008; Brandan and Gutierrez, 2013; Jan et al., 2016). Mouse knockouts for *Dcn*, *Lum*, *Fmod*, and *Biglycan* (*Bgn*) have phenotypes associated with disrupted collagen fibrillogenesis (Ameye et al., 2002; Chen et al., 2010; Kalamajski and Oldberg, 2010; Merline et al., 2009). These knockout mice phenotypes are consistent with SLRPs determining the architecture and the mechanical and chemical properties of the ECM. Abnormalities in limb musculature have not been described in these SLRP mouse mutants. A possible explanation is that several SLRPs have redundant or partially redundant roles in ICT, similar to that described for SLRPs *Dcn* and *Bgn* acting in the skin and cornea. Because we have shown that defective MCT affects muscle morphogenesis (Figure 4; Hasson et al., 2010), it is potentially significant that MCT is rich in this family of matrix-modifying proteins. Strikingly, the SLRPs we identified are not expressed uniformly throughout the ICT but in distinct, partially overlapping domains, suggesting a particular signature of SLRP expression may provide a pattern/cue to the nascent muscle bundle tissue precursors through local modulation of signaling pathways and/or ECM matrix. Although our results do not ascribe functional significance to SLRPs, they identify SLRPs at minimum as useful MCT/ICT markers and reveal a pattern or regionalization within the MCT/ICT that could define the territories where distinct muscle bundle primordia will form. The potential existence of MCT subdomains has been proposed (Orgeur et al., 2018; Sefton and Kardon, 2019). Our study provides the first molecular distinction that MCT/ICT subdomains do exist around the nascent limb musculature. This is particularly evident in the expression patterns of *Ker* (class II), *Lum* (class II), and *Dcn* (class I) and the highly restricted pattern of *Epyc* (class III) (Figure 6). SLRPs from the same class compete for the same binding site on collagen (Chen and Birk, 2013); therefore, these unique and dynamic SLRP expression domains could be functionally relevant (Figure 6; Figure S4). Although, the differential expression or functions of SLRPs have not been reported for the limb, the importance of unique combinations of SLRPs in generating transparency of the cornea has been described (Carlson et al., 2005; Chakravarti et al., 1998; Chen et al., 2014). By analogy to their function in cornea, it is possible that different combinations of SLRPs expressed in distinct MCT/ICT domains surrounding nascent muscle bundles modulate ECM content around muscle precursors and, thereby, influence local cellular behavior (Thorsteinsdóttir et al., 2011) and individual muscle bundle formation. In addition to affecting the physical architecture of the ECM, SLRPs could also affect the movement and presentation of secreted signaling molecules in the ECM,

thereby altering the cellular micro-environment and influencing muscle precursor proliferation, migration, and differentiation (Chen and Birk, 2013; Chen et al., 2010, 2014; Schaefer and Iozzo, 2008; Brandan et al., 2008; Dellett et al., 2012; Lorda-Diez et al., 2014; Nikitovic et al., 2012). SLRPs are known to interact with various cytokines and cell surface receptors, including, but not limited to, BMP4, TGF-β, IGF-IR, and integrin α2β1 (Merline et al., 2009; Schaefer and Iozzo, 2008). Our current results do not distinguish whether one or both of these mechanisms are significant in the effect of MCT on muscle bundle formation. However, alterations in the expression pattern of SLRPs in the *Tbx5* mutant limbs (Figure 7) are consistent with them having roles in limb soft tissue morphogenesis and disruption of their activity contributing to soft tissue defects.

STAR★METHODS

Detailed methods are provided in the online version of this paper and include the following:

- KEY RESOURCES TABLE
- LEAD CONTACT AND MATERIALS AVAILABILITY
- EXPERIMENTAL MODEL AND SUBJECT DETAILS
- METHOD DETAILS
  - Transgenic mice and embryos
  - Genotyping
  - FACS and Cell Culture
  - RNA In Situ Hybridization
  - Immunohistochemistry - Optical Projection Tomography and confocal analysis
  - Basic Whole Mount Immunohistochemistry staining protocol
  - Modifications for Whole mount Immunoflourescence staining
  - Mounting of whole limbs for confocal scanning
  - Clearing whole embryos with clearT
  - Determination of muscle orientation values
  - RNA sequencing and *In Silico* analysis
- QUANTIFICATION AND STATISTICAL ANALYSIS
- DATA AND CODE AVAILABILITY

SUPPLEMENTAL INFORMATION

Supplemental Information can be found online at https://doi.org/10.1016/j.celrep.2020.02.037.

ACKNOWLEDGMENTS

We thank Graham Preece and the Flow Cytometry Facility, Jack Waterford, and Biological Services at MRC-NIMR, Mill Hill. Financial support was from the Bettencourt Schueller Foundation, France (to L.B.) and an Erasmus fellowship (European Union) (to M.L.). This work was funded by MRC, United Kingdom grants MC.PC.13052 and MR/S000038/1.

AUTHOR CONTRIBUTIONS

L.B., C.J.S., E.F., and M.L. conducted the experiments. M.H. carried out the Central Moments analysis. S.W. carried out the OPT analysis. D.B. helped with confocal microscopy. A.K. assisted with line maintenance and TwistAmp genotyping. L.B. and M.P.O.L. conceived and designed the experiments. L.B., C.J.S., and M.P.O.L. wrote the paper.

DECLARATION OF INTERESTS

The authors declare no competing interests.

Received: September 25, 2017
Revised: January 15, 2020
Accepted: February 7, 2020
Published: March 10, 2020

REFERENCES

Abmayr, S.M., and Pavlath, G.K. (2012). Myoblast fusion: lessons from flies and mice. Development *139*, 641–656.

Ameye, L., Aria, D., Jepsen, K., Oldberg, A., Xu, T., and Young, M.F. (2002). Abnormal collagen fibrils in tendons of biglycan/fibromodulin-deficient mice lead to gait impairment, ectopic ossification, and osteoarthritis. FASEB J. *16*, 673–680.

Baldock, R., Bard, J., Brune, R., Hill, B., Kaufman, M., Opstad, K., Smith, D., Stark, M., Waterhouse, A., Yang, Y., and Davidson, D. (2001). The Edinburgh Mouse Atlas: using the CD. Brief. Bioinform. *2*, 159–169.

Basson, C.T., Bachinsky, D.R., Lin, R.C., Levi, T., Elkins, J.A., Soults, J., Grayzel, D., Kroumpouzou, E., Traill, T.A., Leblanc-Straceski, J., et al. (1997). Mutations in human TBX5 [corrected] cause limb and cardiac malformation in Holt-Oram syndrome. Nat. Genet. *15*, 30–35.

Brandan, E., and Gutierrez, J. (2013). Role of skeletal muscle proteoglycans during myogenesis. Matrix Biol. *32*, 289–297.

Brandan, E., Cabello-Verrugio, C., and Vial, C. (2008). Novel regulatory mechanisms for the proteoglycans decorin and biglycan during muscle formation and muscular dystrophy. Matrix Biol. *27*, 700–708.

Bruneau, B.G., Nemer, G., Schmitt, J.P., Charron, F., Robitaille, L., Caron, S., Conner, D.A., Gessler, M., Nemer, M., Seidman, C.E., and Seidman, J.G. (2001). A murine model of Holt-Oram syndrome defines roles of the T-box transcription factor Tbx5 in cardiogenesis and disease. Cell *106*, 709–721.

Buckingham, M., and Vincent, S.D. (2009). Distinct and dynamic myogenic populations in the vertebrate embryo. Curr. Opin. Genet. Dev. *19*, 444–453.

Buckingham, M., Bajard, L., Chang, T., Daubas, P., Hadchouel, J., Meilhac, S., Montarras, D., Rocancourt, D., and Relaix, F. (2003). The formation of skeletal muscle: from somite to limb. J. Anat. *202*, 59–68.

Carlson, E.C., Liu, C.Y., Chikama, T., Hayashi, Y., Kao, C.W., Birk, D.E., Funderburgh, J.L., Jester, J.V., and Kao, W.W. (2005). Keratocan, a cornea-specific keratan sulfate proteoglycan, is regulated by lumican. J. Biol. Chem. *280*, 25541–25547.

Casar, J.C., McKechnie, B.A., Fallon, J.R., Young, M.F., and Brandan, E. (2004). Transient up-regulation of biglycan during skeletal muscle regeneration: delayed fiber growth along with decorin increase in biglycan-deficient mice. Dev. Biol. *268*, 358–371.

Chakravarti, S., Magnuson, T., Lass, J.H., Jepsen, K.J., LaMantia, C., and Carroll, H. (1998). Lumican regulates collagen fibril assembly: skin fragility and corneal opacity in the absence of lumican. J. Cell Biol. *141*, 1277–1286.

Chen, S., and Birk, D.E. (2013). The regulatory roles of small leucine-rich proteoglycans in extracellular matrix assembly. FEBS J. *280*, 2120–2137.

Chen, S., Oldberg, A., Chakravarti, S., and Birk, D.E. (2010). Fibromodulin regulates collagen fibrillogenesis during peripheral corneal development. Dev. Dyn. *239*, 844–854.

Chen, S., Young, M.F., Chakravarti, S., and Birk, D.E. (2014). Interclass small leucine-rich repeat proteoglycan interactions regulate collagen fibrillogenesis and corneal stromal assembly. Matrix Biol. *35*, 103–111.

Chevallier, A., Kieny, M., and Mauger, A. (1977). Limb-somite relationship: origin of the limb musculature. J. Embryol. Exp. Morphol. *41*, 245–258.

Colasanto, M.P., Eyal, S., Mohassel, P., Bamshad, M., Bonnemann, C.G., Zelzer, E., Moon, A.M., and Kardon, G. (2016). Development of a subset of forelimb muscles and their attachment sites requires the ulnar-mammary syndrome gene Tbx3. Dis. Model. Mech. *9*, 1257–1269.

DeLaurier, A., Schweitzer, R., and Logan, M. (2006). Pitx1 determines the morphology of muscle, tendon, and bones of the hindlimb. Dev. Biol. *299*, 22–34.

Delaurier, A., Burton, N., Bennett, M., Baldock, R., Davidson, D., Mohun, T.J., and Logan, M.P. (2008). The Mouse Limb Anatomy Atlas: an interactive 3D tool for studying embryonic limb patterning. BMC Dev. Biol. *8*, 83.

Dellett, M., Hu, W., Papadaki, V., and Ohnuma, S. (2012). Small leucine rich proteoglycan family regulates multiple signalling pathways in neural development and maintenance. Dev. Growth Differ. *54*, 327–340.

Fadic, R., Mezzano, V., Alvarez, K., Cabrera, D., Holmgren, J., and Brandan, E. (2006). Increase in decorin and biglycan in Duchenne Muscular Dystrophy: role of fibroblasts as cell source of these proteoglycans in the disease. J. Cell. Mol. Med. *10*, 758–769.

Gao, Y., Lan, Y., Liu, H., and Jiang, R. (2011). The zinc finger transcription factors Osr1 and Osr2 control synovial joint formation. Dev. Biol. *352*, 83–91.

Grim, M., and Wachtler, F. (1991). Muscle morphogenesis in the absence of myogenic cells. Anat. Embryol. (Berl.) *183*, 67–70.

Hasson, P., DeLaurier, A., Bennett, M., Grigorieva, E., Naiche, L.A., Papaioannou, V.E., Mohun, T.J., and Logan, M.P. (2010). Tbx4 and tbx5 acting in connective tissue are required for limb muscle and tendon patterning. Dev. Cell *18*, 148–156.

Ivanova, A., Signore, M., Caro, N., Greene, N.D., Copp, A.J., and Martinez-Barbera, J.P. (2005). *In vivo* genetic ablation by Cre-mediated expression of diphtheria toxin fragment A. Genesis *43*, 129–135.

Jan, A.T., Lee, E.J., and Choi, I. (2016). Fibromodulin: A regulatory molecule maintaining cellular architecture for normal cellular function. Int. J. Biochem. Cell Biol. *80*, 66–70.

Kalamajski, S., and Oldberg, A. (2010). The role of small leucine-rich proteoglycans in collagen fibrillogenesis. Matrix Biol. *29*, 248–253.

Kardon, G. (1998). Muscle and tendon morphogenesis in the avian hind limb. Development *125*, 4019–4032.

Kardon, G. (2011). Development of the musculoskeletal system: meeting the neighbors. Development *138*, 2855–2859.

Kardon, G., Campbell, J.K., and Tabin, C.J. (2002). Local extrinsic signals determine muscle and endothelial cell fate and patterning in the vertebrate limb. Dev. Cell *3*, 533–545.

Kardon, G., Harfe, B.D., and Tabin, C.J. (2003). A Tcf4-positive mesodermal population provides a prepattern for vertebrate limb muscle patterning. Dev. Cell *5*, 937–944.

Kuwajima, T., Sitko, A.A., Bhansali, P., Jurgens, C., Guido, W., and Mason, C. (2013). ClearT: a detergent- and solvent-free clearing method for neuronal and non-neuronal tissue. Development *140*, 1364–1368.

Lan, Y., Wang, Q., Ovitt, C.E., and Jiang, R. (2007). A unique mouse strain expressing Cre recombinase for tissue-specific analysis of gene function in palate and kidney development. Genesis *45*, 618–624.

Lee, A.S., Harris, J., Bate, M., Vijayraghavan, K., Fisher, L., Tajbakhsh, S., and Duxson, M. (2013). Initiation of primary myogenesis in amniote limb muscles. Dev. Dyn. *242*, 1043–1055.

Lee, E.J., Jan, A.T., Baig, M.H., Ashraf, J.M., Nahm, S.S., Kim, Y.W., Park, S.Y., and Choi, I. (2016). Fibromodulin: a master regulator of myostatin controlling progression of satellite cells through a myogenic program. FASEB J. *30*, 2708–2719.

Li, Q.Y., Newbury-Ecob, R.A., Terrett, J.A., Wilson, D.I., Curtis, A.R., Yi, C.H., Gebuhr, T., Bullen, P.J., Robson, S.C., Strachan, T., et al. (1997). Holt-Oram syndrome is caused by mutations in TBX5, a member of the Brachyury (T) gene family. Nat. Genet. *15*, 21–29.

Lobe, C.G., Koop, K.E., Kreppner, W., Lomeli, H., Gertsenstein, M., and Nagy, A. (1999). Z/AP, a double reporter for cre-mediated recombination. Dev. Biol. *208*, 281–292.

Lorda-Diez, C.I., García-Porrero, J.A., Hurlé, J.M., and Montero, J.A. (2014). Decorin gene expression in the differentiation of the skeletal connective tissues of the developing limb. Gene Expr. Patterns *15*, 52–60.

Mathew, S.J., Hansen, J.M., Merrell, A.J., Murphy, M.M., Lawson, J.A., Hutcheson, D.A., Hansen, M.S., Angus-Hill, M., and Kardon, G. (2011). Connective tissue fibroblasts and Tcf4 regulate myogenesis. Development *138*, 371–384.

Merline, R., Lazaroski, S., Babelova, A., Tsalastra-Greul, W., Pfeilschifter, J., Schluter, K.D., Gunther, A., Iozzo, R.V., Schaefer, R.M., and Schaefer, L. (2009). Decorin deficiency in diabetic mice: aggravation of nephropathy due to overexpression of profibrotic factors, enhanced apoptosis and mononuclear cell infiltration. J. Physiol. Pharmacol. *60*, 5–13.

Minguillon, C., Del Buono, J., and Logan, M.P. (2005). Tbx5 and Tbx4 are not sufficient to determine limb-specific morphologies but have common roles in initiating limb outgrowth. Dev. Cell *8*, 75–84.

Murphy, M., and Kardon, G. (2011). Origin of vertebrate limb muscle: the role of progenitor and myoblast populations. Curr. Top. Dev. Biol. *96*, 1–32.

Murphy, M.M., Lawson, J.A., Mathew, S.J., Hutcheson, D.A., and Kardon, G. (2011). Satellite cells, connective tissue fibroblasts and their interactions are crucial for muscle regeneration. Development *138*, 3625–3637.

Nikitovic, D., Aggelidakis, J., Young, M.F., Iozzo, R.V., Karamanos, N.K., and Tzanakakis, G.N. (2012). The biology of small leucine-rich proteoglycans in bone pathophysiology. J. Biol. Chem. *287*, 33926–33933.

Orgeur, M., Martens, M., Leonte, G., Nassari, S., Bonnin, M.A., Börno, S.T., Timmermann, B., Hecht, J., Duprez, D., and Stricker, S. (2018). Genome-wide strategies identify downstream target genes of chick connective tissue-associated transcription factors. Development *145*, dev161208.

Pryce, B.A., Brent, A.E., Murchison, N.D., Tabin, C.J., and Schweitzer, R. (2007). Generation of transgenic tendon reporters, ScxGFP and ScxAP, using regulatory elements of the scleraxis gene. Dev. Dyn. *236*, 1677–1682.

Riddle, R.D., Johnson, R.L., Laufer, E., and Tabin, C. (1993). Sonic hedgehog mediates the polarizing activity of the ZPA. Cell *75*, 1401–1416.

Rodriguez-Guzman, M., Montero, J.A., Santesteban, E., Gañan, Y., Macias, D., and Hurle, J.M. (2007). Tendon-muscle crosstalk controls muscle bellies morphogenesis, which is mediated by cell death and retinoic acid signaling. Dev. Biol. *302*, 267–280.

Schaefer, L., and Iozzo, R.V. (2008). Biological functions of the small leucine-rich proteoglycans: from genetics to signal transduction. J. Biol. Chem. *283*, 21305–21309.

Schroeter, S., and Tosney, K.W. (1991a). Spatial and temporal patterns of muscle cleavage in the chick thigh and their value as criteria for homology. Am. J. Anat. *191*, 325–350.

Schroeter, S., and Tosney, K.W. (1991b). Ultrastructural and morphometric analysis of the separation of two thigh muscles in the chick. Am. J. Anat. *191*, 351–368.

Sefton, E.M., and Kardon, G. (2019). Connecting muscle development, birth defects, and evolution: An essential role for muscle connective tissue. Curr. Top. Dev. Biol. *132*, 137–176.

Spornitz, U.M. (1978). Studies on the liver of xenopus laevis. III. The ultrastructure and the glycogen content of the developing liver. Anat. Embryol. (Berl.) *154*, 1–25.

Srinivas, S., Watanabe, T., Lin, C.S., William, C.M., Tanabe, Y., Jessell, T.M., and Costantini, F. (2001). Cre reporter strains produced by targeted insertion of EYFP and ECFP into the ROSA26 locus. BMC Dev. Biol. *1*, 4.

Stricker, S., Brieske, N., Haupt, J., and Mundlos, S. (2006). Comparative expression pattern of Odd-skipped related genes Osr1 and Osr2 in chick embryonic development. Gene Expr. Patterns *6*, 826–834.

Swinehart, I.T., Schlientz, A.J., Quintanilla, C.A., Mortlock, D.P., and Wellik, D.M. (2013). Hox11 genes are required for regional patterning and integration of muscle, tendon and bone. Development *140*, 4574–4582.

Thorsteinsdóttir, S., Deries, M., Cachaço, A.S., and Bajanca, F. (2011). The extracellular matrix dimension of skeletal muscle development. Dev. Biol. *354*, 191–207.

Tozer, S., Bonnin, M.A., Relaix, F., Di Savino, S., García-Villalba, P., Coumailleau, P., and Duprez, D. (2007). Involvement of vessels and PDGFB in muscle splitting during chick limb development. Development *134*, 2579–2591.

Vallecillo-García, P., Orgeur, M., Vom Hofe-Schneider, S., Stumm, J., Kappert, V., Ibrahim, D.M., Börno, S.T., Hayashi, S., Relaix, F., Hildebrandt, K., et al. (2017). Odd skipped-related 1 identifies a population of embryonic fibro-adipogenic progenitors regulating myogenesis during limb development. Nat. Commun. *8*, 1218.

Zanotti, S., Negri, T., Cappelletti, C., Bernasconi, P., Canioni, E., Di Blasi, C., Pegoraro, E., Angelini, C., Ciscato, P., Prelle, A., et al. (2005). Decorin and biglycan expression is differentially altered in several muscular dystrophies. Brain *128*, 2546–2555.

Zhou, J., Gao, Y., Zhang, Z., Zhang, Y., Maltby, K.M., Liu, Z., Lan, Y., and Jiang, R. (2011). Osr2 acts downstream of Pax9 and interacts with both Msx1 and Pax9 to pattern the tooth developmental field. Dev. Biol. *353*, 344–353.

STAR★METHODS

KEY RESOURCES TABLE

REAGENT or RESOURCE	SOURCE	IDENTIFIER
Antibodies		
My32 (Fast Skeletal muscle myosin ab)	Sigma	M4276
MF20 (Myosin Heavy Chain)	DSHB	RRID:AB_2147781
F59 (Myosin Heavy chain)	DSHB	RRID:AB_528373
MyoD	DAKO	Cat M3512; RRID:AB_2148874
Digoxygenin-AP	Roche	11093274910
Sox9	R&D	Cat AF3075; RRID:AB_2194160
Tcf4	Cell signalling	Cat C48H11; RRID:AB_2199816
GFP	BioRad	4745-1051
GFP	Thermofisher	Cat A-21311; RRID:AB_221477
Donkey anti-sheep Alexa 488	Thermofisher	Cat A-11015; RRID:AB_2534082
Biotin-SP donkey anti-rabbit	Jackson ImmunoRes	Cat 711-065-152; RRID:AB_2340593
Streptavidin conjugated Cy3	Jackson ImmunoRes.	Cat 016-160-084; RRID:AB_2337244
Goat anti-mouse Alexa 555	LifeTechnologies	Cat A21422; RRID:AB_141822
Chemicals, Peptides, and Recombinant Proteins		
Focus Clear	Cell Explorers Lab Co.	FC-102
Critical Commercial Assays		
TwistAmpR exo system	TwistDx UK	*TAEXO02KIT*
QiaShredder	Qiagen	Cat No./ID: 79654
Corning CellBIND surface tissue culture dish	Corning	3294
RNAeasy MIni Kit	Qiagen	Cat No./ID: 74104
Tru seq RNA sample preparation kit	Illumina	RS-122-2001
Deposited Data		
The FastQ files of the RNA seq data generated in this study are available at ArrayExpress.	ArrayExpress https://www.ebi.ac.uk/arrayexpress/	accession E-MTAB-8772
Experimental Models: Organisms/Strains		
Tbx5flox	Bruneau et al., 2001	Seidman lab
Osr2IRESCre	Lan et al., 2007	Stricker lab
ROSAYFP	Srinivas et al., 2001	Costantini lab
ROSAeGFP-DTA	Ivanova et al., 2005	Martinez-Barbera lab
Z/AP	Lobe et al., 1999	Nagy lab
ScxGFP	Pryce et al., 2007	Schweitzer lab
Oligonucleotides		
**Tbx5loxsiteFWD:** ATAACTTCGTATAATGTATGCTATAC GAGT[T(TAMRA)]HTC[T(BHQ-2)] AGTTGTGTGCCTTC	TwistDx UK	n/a
**Tbx5WTsiteFWDFAM** CGAGGTATGGGGGAGCCGAGTTC TGTACTAGT[T(FAM)]HTG[T(BHQ-1)] GCCTTCAGCTTTC	TwistDx UK	n/a
Recombinant DNA		
Dcn (Image clone 40130798	Source Biosciences	https://www.sourcebioscience.com/home
Kera (Image clone 40046884	Source Biosciences	https://www.sourcebioscience.com/home

(*Continued on next page*)

Continued

REAGENT or RESOURCE	SOURCE	IDENTIFIER
Lum (Image clone 3582135)	Source Biosciences	https://www.sourcebioscience.com/home
Epyc, (Image clone 4037028	Source Biosciences	https://www.sourcebioscience.com/home
Fmod (Image clone 30058603<br>*Ogn* (Image clone 5067073)	Source Biosciences	https://www.sourcebioscience.com/home
Software and Algorithms		
Avadis NGS	Strand	n/a
Fiji-Image J	Image-J.net	n/a
Wolfram Mathematica	Champaign Il USA	n/a

LEAD CONTACT AND MATERIALS AVAILABILITY

Further information and requests for resources and reagents should be directed to and will be fulfilled by the Lead Contact, Malcolm P.O. Logan (malcolm.logan@kcl.ac.uk)

All unique/stable reagents generated in this study are available from the Lead Contact with a completed Materials Transfer Agreement

EXPERIMENTAL MODEL AND SUBJECT DETAILS

All regulated work using animal model (mouse) was carried out under the appropriate UK Home Office Animal (Scientific Procedures) Project Licence (Holder: Malcolm P.O. Logan) and was reviewed and approved internally through the local Ethical Review Panels (ERP) at King's College London.

Tbx5$^{lox/lox}$ strain is originally described in Bruneau et al. (2001).

ROSA26YFP (Gt(ROSA)26Sor$^{tm1(EYFP)Cos}$) reporter transgenic is originally described in Srinivas et al. (2001).

Osr2^{IREScre} (Osr2$^{tm2(cre)Jian}$) is originally described in Lan et al. (2007).

ScxGFP strain is originally reported in Pryce et al. (2007).

Z/AP (CAG-Bgeo/ALPP)1Lbe) reporter transgenic is originally described in Lobe et al. (1999).

ROSA-eGFP-DTA (Gt(ROSA)26Sor$^{tm1(DTA)Jpmb}$) is originally described in Ivanova et al.(2005).

METHOD DETAILS

Transgenic mice and embryos

Mouse embryos were staged according to Kaufman (Baldock et al., 2001) and the web tool https://dmdd.org.uk/. Noon on the day a vaginal plug was observed was taken as E0.5 day gestation. The mouse lines used have been described previously; *Tbx5* (Bruneau et al., 2001), *Rosa26YFP* (Srinivas et al., 2001) *Osr2^{IRESCre}* (Lan et al., 2007), *ScxGFP* (Pryce et al., 2007), *Z/AP* (Lobe et al., 1999), *ROSA26-eGFP-DTA* (Ivanova et al., 2005). *Tbx5 $^{lx/+}$;Osr2Cre* heterozygotes are viable and fertile and were used as controls for comparison with *Tbx5$^{lx/lx}$;Osr2Cre* mutants. A minimum of 3 limbs were analysed for each condition.

Genotyping

Lox, wild-type and *Cre* alleles were identified by conventional PCR genotyping as previously described (Minguillon et al., 2005) and using the TwistAmp^{R} exo system (TwistDx, UK) following the manufacturer's instructions. Primers used for Twist amplification were:

Tbx5loxsiteFWD ATAACTTCGTATAATGTATGCTATACGAGT[T(TAMRA)]HTC[T(BHQ-2)]AGTTGTGTGCCTTC

Tbx5WTsiteFWDFAM CGAGGTATGGGGGAGCCGAGTTCTGTACTAGT[T(FAM)]HTG[T(BHQ-1)]GCCTTCAGCTTTC

The presence of *ScxGFP* and *Osr2Cre;RosaYFP* transgenes was identified by examination of the limbs under fluorescent light.

FACS and Cell Culture

Tbx5$^{lx/+}$;Osr2Cre;RosaYFP and *Tbx5$^{lx/lx}$;Osr2Cre;RosaYFP* embryos were harvested in cold DMEM/F12-10%FBS-1%glutamax. Genotyping of each embryo was performed using the TwistAmp^{R} exo system after digestion of tissue for 20 min at 95°C. Forelimbs were collected and cells dissociated in collagenase/dispase 0.5mg/ml. Cells suspensions were sorted to obtain YFP positive and YFP negative cells fractions by FACS at 4°C using a BD Influx cell sorter (laser 488nm) at 32psi sample pressure, 30 psi sheath fluid, 86 microns nozzle. Both fractions were collected in DMEM/F12-10%FBS. For culture, cells were plated on Corning^{R}CellBIND^{R}Surface plates in DMEM/10%FBS/1%L-Glutamine/1%Pen-strep.

RNA In Situ Hybridization

Whole-mount and section in situ hybridization were carried out essentially as previously described (Riddle et al., 1993). A minimum of three mutant embryos were analysed at each stage described with each probe. *Pax3, MyoD*, *Myog*, *Scx* (Hasson et al., 2010), *Dcn* (Image clone 40130798), *Kera* (Image clone 40046884), *Lum* (Image clone 3582135), *Epyc*, (Image clone 4037028), *Fmod* (Image clone 30058603), and *Ogn* (Image clone 5067073).

The protocol on cryosections was carried out essentially as described by Riddle et al. (1993) with the following amendments to detect mRNA trancripts.

- leave the slides dry on the bench for 30 minutes, RT.
- 30 min in PBS1X (in a clean rack, washed before with detergent and RNAse easy).
- 10 min in PFA4% then 2x5min in PBS
- prehybrydisation 2h at RT in a humidified box soaked in SSC1X-50%formamide (from SSC20X pH7), 500 μl prehybridization solution per slide.
- in a tube, mix 100μl of prehyb solution + 1-3 μl of the probe and put it at 80°C for 10min, then immediately on ice for 5min.
- remove prehyb solution from the slide
- spread the hybridisation solution (prehyb + probe) on the slide and cover with RNAse free glass coverslip.
- incubate O/N 70°C in a hybridisation oven, no shaking, in a sealed humidified box (made wet with 1xSSC-50% formamide).

washes :

- 1 X in 50%formamide;1xSSC;0.1% Tween at 65°C to remove the coverslips (in a large volume, pull the slide with forceps, the coverslips usually fall down themselves with the heat but if not, help them pushing gently towards the bottom of the slide)

Then further washes as follows:

- 2x30min in 50%formamide-1xSSC-0.1%tween at 65°C.
- 2x30min in Maleic acid buffer, RT.

blocking :

- 2h at RT in 2%BBR(Boehringer Blocking reagent-Roche)-20%SS-maleic acid buffer. 500μl/slide

Incubation with anti-dig :

- 1/3000 dilution in 2%BBR-20%Sheep serum in maleic acid buffer, 100μl/slide covered with a piece of parafilm.
- incubate O/N at 4°C in a humidified chamber with water.

washes :

- 4x30min in maleic buffer
- 2x15min in NTMT

detection :

- either in a large volume OR with 400-500μl/slide of detection solution, in the dark : 1ml NTMT + 3,4μl NBT (0.075g/ml in 70& DMF + 3.85μl BCIP(Na salt) (0.05 mg/ml in H2O)

can last few hours or several days, depending on the probe.

- once the staining is satisfactory, rinse in NTMT then PBT several times, then quickly in water and mounted in aqueous mounting medium.
- if the staining needs several days, leave the slides in NTMT O/N at 4°C at the end of each day.

Solutions :

prehyb solution : 50% formamide, 5X SSC (3M NaCl; 0.3M NaCitrate), pH 4.5, 1% Sodium dodecyl sulphate (SDS)
maleic acid buffer : 400ml of maleic acid 250mM pH7.2 + 30ml NaCl (5M) + 10ml 10%Tween.

Immunohistochemistry - Optical Projection Tomography and confocal analysis

Immunohistochemistry were performed on 12 microns sections of *Osr2Cre;RosaYFP* embryos. Sections were left at room temperature for 30 minutes then rinsed in PBS for 1h, blocked for 2h in PBS-1%BSA-10%NGS, incubated overnight in primary antibodies at 4°C. Sections were then washed quickly in PBS, incubated 2h in the same blocking solution with secondary antibodies and DAPI (1:2000) and finally washed several times in PBS before mounting in PBS-50%glycerol. Confocal images were produced using

the Zeiss LSM5 Pascal. Whole-mount immunostainings and OPT analyses were done as previously described (DeLaurier et al., 2006). Whole forelimbs were cleared after staining in 100% glycerol or Focus Clear reagent according to manufacturers' instructions (CelExplorer lab) and then mounted. Confocal images were produced using either the Zeiss LSM5 Pascal or Leica TCS Sp5 (objective 63x/1.3NA Glycerol (Leica HCX PL APO CS 63x /1.3 GLYC (s/n 11506194)). Identification of skeletal elements, muscles and tendons was done using the mouse limb anatomy atlas (Delaurier et al., 2008).

The following antibodies were used: mouse anti-skeletal myosin (my32; 1:800; Sigma), mouse anti-myosin heavy chain and sarcomere myosin (F59 & MF20; both 1:50 DSHB), mouse anti-MyoD (Dako 1:50 for whole mount, 1:200 for sections), sheep anti-digoxygenin (Roche, 1:3000), rabbit anti-Myogenin (5FD, DSHB, 1:10), rabbit anti-GFP (Invitrogen A21311, 1:250), sheep anti-GFP (AbD Serotec, 1:200) and donkey anti-sheep alexa-488 (Invitrogen 1:300), mouse anti-Sox9 (R&D systems, 1:200), rabbit anti-Tcf4 (Clone C48H11 Cell Signalling Technology 2569, 1:50), Biotin-SP donkey anti-rabbit (Jackson ImmunoResearch 711-065-152, 1:500), Streptavidin conjugated Cy3 (Jackson ImmunoResearch 016-160-084, 1:200), and Alexa Fluor 555 goat anti-mouse IgG (LifeTechnologies A21422, 1:400).

Basic Whole Mount Immunohistochemistry staining protocol

To obtain deep penetration of antibody staining in whole mount preparation of limbs we used a slightly modified protocol including DMSO to increase antibody penetration.

Embryos fixed in 4% Paraformaldehyde (PFA).

Embryos were stored in Methanol (100%) before processing.

Limbs are removed from the embryo and (if older than E13.5) skinned (in 100% methanol solution) prior to the staining.

Day 1:

1. Rehydrate limb in 50% Methanol:Phosphate-buffered saline; 0.1% Tween (PBT) 1 x 10 min, RT, rocking. (if not fixed previously in Methanol - go to 50% Methanol/PBT to 100% Methanol for 1h wash then back to 50% and finally PBT)
2. Wash limb in PBT 3 x 5 min, RT, rocking.
3. Incubate limbs in PBT, 1 hr, 70^0C, rocking (to inactivate endogenous Alkaline phosphatase (AP)). This step only for AP staining. Omit if fluorescence staining.)
4. Bleach with 6% Hydrogen Peroxide in PBT, 1hr RT, rocking. (Omit if fluorescence staining.)
5. wash in PBT 3 x 5 min. (only necessary if steps 3 and 4 above were carried out)
6. Block 1 hr (Block solution: 1 x PBS; 0.1% Triton; 1% BSA; 0.15% glycine), RT, rocking.
7. antibodies anti myosin : F59 and MF20 both at 1/50 in block solution O/N 4^0C, rocking

Day 2:

1. wash in PBT 3 x 5 min, RT, rocking.
2. wash in PBT 3 x 1 hr, RT, rocking.
3. Block 1 hr (Block solution: 1 x PBS; 0.1% Triton; 1% BSA; 0.15% glycine), RT, rocking.
4. Incubate antibody anti-mouse-AP (Fc portion) (1:800) + anti-myosin-AP (My32) (1:800) in block solution, overnight, 4^0C rocking.

Day 3:

1. wash antibody in TBST 3 x 5 min, RT, rocking.
2. wash antibody in TBST 5 x 1 hr, RT, rocking.
3. leave overnight in TBST (4^0C) or proceed to detection.

Detection: (AP only)

1. wash 3 x 5 min in NTMT, RT, rocking.
2. Incubate with fresh NBT and BCIP.
3. cover tubes and leave rocking in RT – AP staining is usually visible within 3 minutes but requires longer to go to completion.

postfix: 4% PFA; 0.2% glutaraldehyde.

Solutions

PBS (137mM NaCl, 2.7mM KCl, 10mM Na2HPO4, 1.8 mM KH2PO4)
TBST (0.14M NaCl, 2.7mM KCl, 25mMTrisHCl, pH7.5 1% Tween-20)
NTMT (100mM NaCl, 100mM TrisHCl, pH 9.5, 50mM MgCl2, 0.1% Tween-20)

Modifications for Whole mount Immunoflourescence staining

Post-fix/permeabilization samples in Methanol:DMSO (4:1) for 2- 4 weeks.
Block solution: 1 x PBS; 5% goat serum, 20% DMSO
Incubate primary Abs for overnight or up to 1 week at 4°C.

Mounting of whole limbs for confocal scanning

To confocal scan the relatively large limb specimens we mounted processed samples on slides in clearing agents.
Stained limbs were placed directly onto a Superfrost slide with a transfer pipette
The majority of the PBS solution was removed
4 drops of petroleum jelly in 4 corners following the dimensions of the coverslip were placed around the sample.

- add 200 μl of Focus Clear on top of the limb
- place a coverslip on top of sample in Focus Clear and gently press flat to create a vaseline seal at the corners. Don't press to much but sufficient pressure so the limb is flattened for the confocal scan
- leave the slide at 4°C O/N taking care to be sure Focus Clear covers the limb
- after scan, the limb can be stored in PBS, keep at 4°C

Clearing whole embryos with clearT

Essentially following the protocol of Kuwajima et al., 2013, Development
After post fixing the immunostaining all steps in the dark:

Remove PBS from samples
Add 25% formamide/10% PEG, 1 hour rocking RT
50% formamide/20% PEG, 1 hour rocking RT
50% formamide/20% PEG, O/N at 4°C

The embryos are ready for mounting and imaging.
Caution : Do not store the embryos in formamide. For storage rinse the sample in PBS and store in fresh PBS (azide can be added).
Solutions :

50% formamide/20% polyethylene glycol (PEG): mix formamide (99.6%, considered 100%) with 40% PEG/H2O (wt/vol) at a ratio of 1:1 (vol/vol).
25% formamide/10% PEG: mix 50% formamide plus 20% PEG/H2O (wt/vol) at a ratio of 1:1 (vol/vol).
40% PEG solution: stir powdered PEG 8000 MW (Sigma-Aldrich) in warm H2O for 30 minutes, (stable at room temperature for several months)

Determination of muscle orientation values

To analyse orientation, myocytes and nascent fibres were labelled using myogenin and myosin antibodies and the dorsal forelimbs were scanned using confocal microscopy. Image files generated were opened in the open source software Fiji-ImageJ2 and a graphic tablet was used to draw a line over the long axis of myogenin and myosin positive elongated cells, for each individual Z plane, in a new overlying layer. Each line represents the orientation vector of the cell that we could assign an angle value. These binary images of manually detected fibres were imported into Wolfram Mathematica (Champaign, IL) where the orientation of fibres were determined using our own bespoke scripts. Briefly, overlaps between fibres, or morphological branch points, were removed before orientation analysis was performed using the second order central moments on a fibre-by-fibre basis. Angles were normalised to a range of 0-to-Pi radians (or 0° to 180°) in 32 different bins and colour-coded according to similar orientation using a Hue look up table.

RNA sequencing and *In Silico* analysis

Sorted cells from E11.5 and E12.5 *Tbx5*$^{lx/+}$*;Osr2Cre;RosaYFP* and *Tbx5*$^{lx/lx}$*;Osr2Cre;RosaYFP* forelimbs were centrifuged at 500g 5min at 4°C. Supernatant was removed and RLT buffer with β-mercaptoethanol was added, transferred to Qiashredder columns and centrifuged 2min at full speed in a bench centrifuge. The cells were then stored at -80°C. RNAs were extracted using the RNeasy Mini Kit (QIAGEN) according to manufacturers' instructions. Each sample is a pool of YFP positive or YFP negative forelimb cells at E11.5 and E12.5. A total of 0.5μg of RNA per sample was used to generate cDNA libraries using the Illumina Truseq RNA sample preparation V2 kit. A single-read sequencing was done, generating 75bp reads. In total, three independent samples of control and mutants cells were analysed for each stage and each YFP+ and YFP- fraction.

QUANTIFICATION AND STATISTICAL ANALYSIS

Avadis NGS software (Strand NGS.com) was used to align the outcome reads and data analysis. Alignment was done against the transcriptome with transcript model "Ensembl Genes and transcript". Quantification was performed on all aligned reads using the DESeq normalization algorithm and genes were filtered by expression with a lower cut off of 10 raw counts. A moderate Student's t-test was applied to identify differentially expressed genes in the different cell populations and to generate the given p values. $p<0.05$ was considered significant and was used as a threshold. Fold change analysis corresponds to the ratio of the read densities between two conditions. Its calculation is the antilog of difference in averaged, normalized values between conditions.

DATA AND CODE AVAILABILITY

The FastQ files of the RNA seq data generated in this study are available at ArrayExpress accession E-MTAB-8772.

Cell Reports, Volume *30*

Supplemental Information

Individual Limb Muscle Bundles Are Formed through Progressive Steps Orchestrated by Adjacent Connective Tissue Cells during Primary Myogenesis

Laurianne Besse, Caroline J. Sheeba, Mark Holt, Maurice Labuhn, Susan Wilde, Eleanor Feneck, Donald Bell, Ania Kucharska, and Malcolm P.O. Logan

Supplemental Text and Figures

Individual limb muscle bundles are formed through progressive steps orchestrated by adjacent connective tissue cells during primary myogenesis.

Besse et al.,

Cell Reports D-17-03107

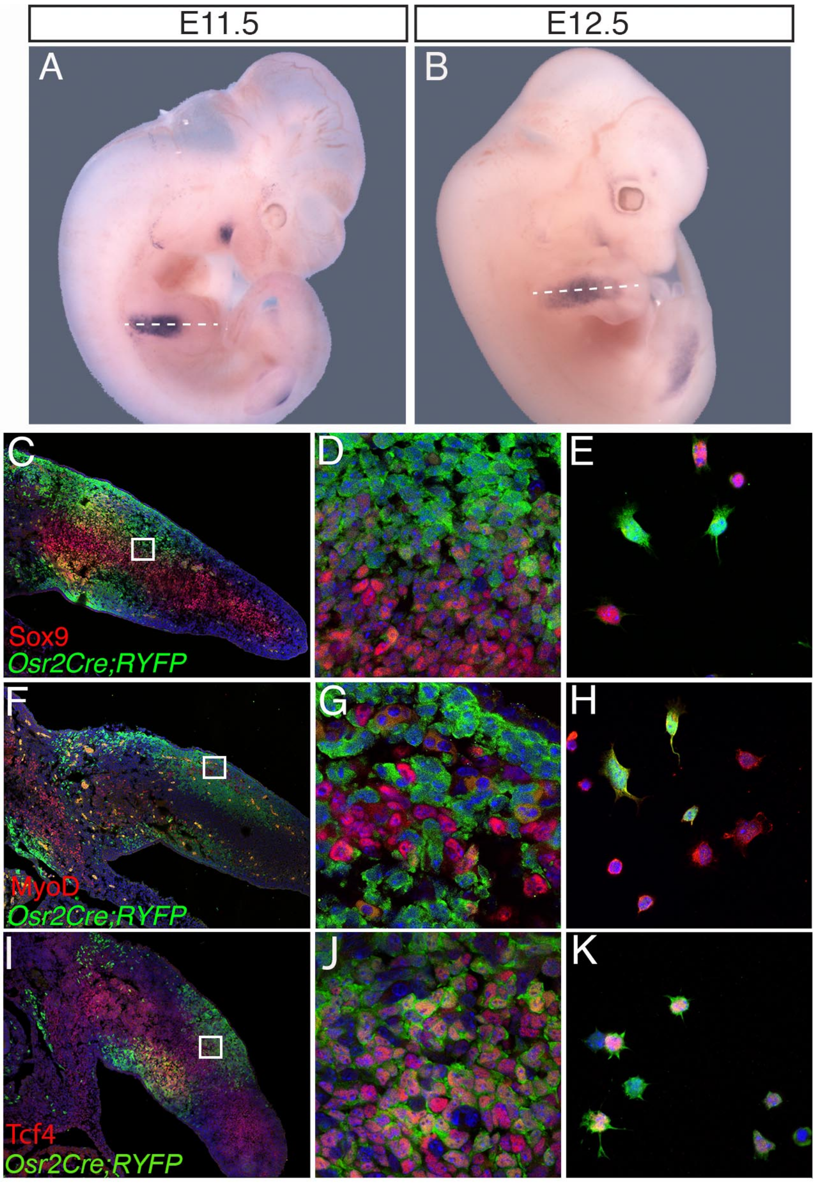

Figure S1. *Osr2Cre* is active in forelimb MCT. Related to Figures 2, 3 and 4
(A, B) Whole mount immunohistochemistry to detect alkaline phosphatase showing Cre activity almost exclusively restricted to the limbs at E11.5 (A) and E12.5 (B). Dashed lines show approximate plane of section shown in C,D, F,G, I,J. Immunohistochemistry on E11.5 *Osr2Cre;RosaYFP* forelimb cryosections to detect Sox9 (C-D), MyoD (F-G) and Tcf4 (I-J) (all red channel) also labelled for YFP (green) and DAPI (blue). The majority of *Osr2Cre* activity (YFP+) was not detected in the cartilage

cells (Sox9 +) or muscle progenitors (MyoD+) precursor cells. *Osr2Cre* activity (YFP+) overlaps with Tcf4+ MCT cells. The same immunohistochemical staining was carried out on dissociated cells in culture isolated from E11.5 limb buds (E, H, K show representative views of labelled cells). E. Of a total of 83 cells analysed, 36 were Sox9+ive only, 41 GFP(cre)+ive only and 6 were double labelled for both GFP(cre) and Sox9. H. Of a total of 431 cells analysed, 315 were MyoD +ive only, 116 were GFP/Cre +ive only and 0 were double-labelled for both GFP(cre) and MyoD. K. Of a total of 116 cells analysed, 5 were TCF+ive only, 46 were GFP/cre+ive only and 65 were double-labelled for TCF4/cre. Panels C,F,I are 20X, panels D-E, G-H and J-K are 100X magnification.

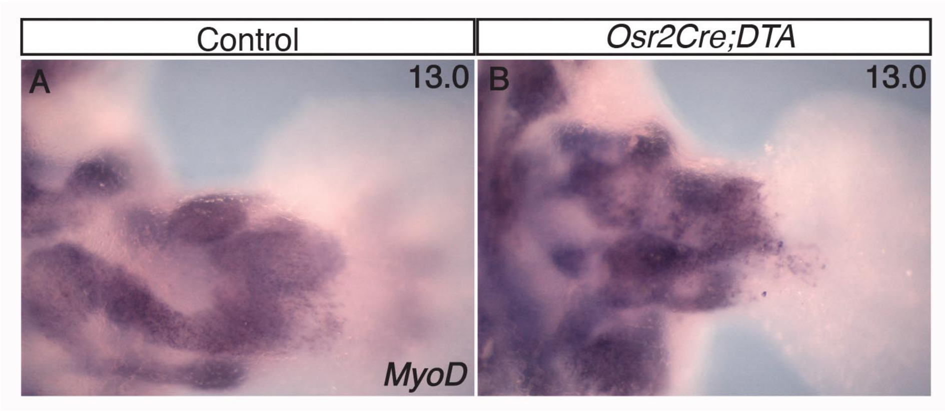

Figure S2. Muscle morphogenesis is disrupted in *ROSA26-eGFP-DTA;Osr2Cre* embryos. Related to Figures 2, 3 and 4
Dorsal view of control (A) and *ROSA26-eGFP-DTA;Osr2Cre* mutant (B) forelimbs at E13.0 processed by whole mount *in situ* hybridisation for *MyoD*. Nascent muscle bundles, aligned fibres and a central zeugopodal domain where *MyoD* expression is excluded can be seen in control (A). In mutant (B) MyoD-positive cells are present but are distributed in an abnormal pattern.

A

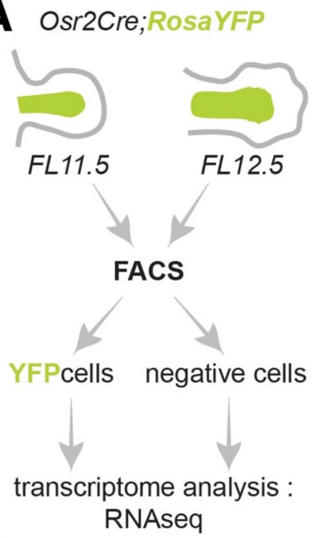

B

Gene	RC +/- 11.5	FC +/- 11.5	p value 11.5	RC +/- 12.5	FC +/- 12.5	p value 12.5
Keratocan	1943.42/58.07	27.06 up	0.0000129	5505.85/206.01	23.2 up	1.35E-04
Epiphycan	167.53/7.73	17.52 up	0.000089	204.05/35.25	7.58 up	0.021
Decorin	956.06/122.14	6.3 up	3.64E-05	6582.79/755.01	7.6 up	0.0037
Fibromodulin	623.25/89.05	5.7 up	6.42E-05	2724.94/633.71	3.7 up	0.001
Osteoglycin	1423.73/203.56	5.7 up	1.25E-04	4539.41/1679.31	2.4 up	0.008
Lumican	5720.96/970.32	4.8 up	6.33E-05	12496.12/2816.32	3.9 up	3.85E-04
Podocan	249.42/44.61	4.52 up	2.93E-04	318.21/124.2	2.2 up	0.0016
Osr2	2888.82/290.12	8.1 up	2.40E-05	1668.51/397.58	3.6 up	9.91E-05
Osr1	3706.34/1043.41	2.9 up	7.08E-05	4329.6/1900.15	2.0 up	0.002

Figure S3. SLRPs are enriched in the limb ICT/MCT population of cells. Related to Figure 6 and Supplementary Figure 4

(A) Schematic diagram of the strategy used to label and isolate ICT cells for transcriptome analysis using the combination of *Osr2Cre* deleter and *ROSAYFP* reporter with FACS to generate YFP+ and YFP- cell pools. (B) Table containing a selected list of some of the most differentially expressed genes comparing the transcriptome of YFP+ ICT/MCT cells with YFP- limb mesenchyme cells.

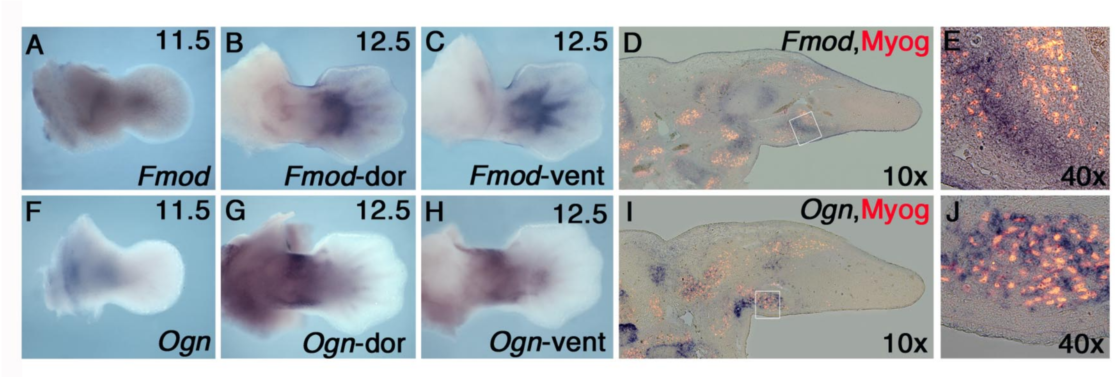

Figure S4. Expression profiles of SLRPs *Fmod* and *Ogn* in the forelimbs. Related to Figure 6. Dorsal view of wild type forelimbs at E11.5 (A,F) and E12.5 (B,G) and the ventral view (C,H) of the E12.5 limbs in B,G, processed for *Fmod* (A-C) and *Ogn* (F-H) by whole mount *in situ* hybridisation. Localisation of SLRP expression with muscle cells detected by section *in situ* hybridisation for *Fmo*d or *Ogn* followed by immunofluorescence for Myogenin at E12.5 and presented as 10X images (D,E). The region boxed in 10X images are 40X magnification (E,J).
